# Finite-Length Spatiotemporal Modelling for Housing Price Network Spillovers

**DOI:** 10.3390/e28050537

**Published:** 2026-05-09

**Authors:** Lu Qiu, Yanzhe Jiao, Gege Dong, Guangcan Cui

**Affiliations:** 1 School of Finance and Business, Shanghai Normal University, Shanghai 200234, China; nuaaqiulu@shnu.edu.cn (L.Q.); 1000553383@smail.shnu.edu.cn (Y.J.); 1000567225@smail.shnu.edu.cn (G.D.); 2 Department of Finance, East China University of Science and Technology, Shanghai 200237, China

**Keywords:** complex network, housing price spillover, nonlinear spatiotemporal integrated model, transfer entropy, minimum spanning tree

## Abstract

Mapping directed spillover pathways in urban housing prices is essential for monitoring the contagion of housing prices across cities. However, existing studies typically rely on either spatial gravity models or time-series models in isolation to analyze intercity connections, thus failing to simultaneously capture the spatiotemporal integration characteristics of housing price contagion. To address this, we embed a finite-length sequence correlation analysis (Correlation-Dependent Balanced Estimation of Diffusion Transfer Entropy, CBEDTE) into the gravity model, yielding the CBEDTE-GM integrated model. Using housing price data from 296 Chinese cities, we construct a spatiotemporal correlation matrix and employ the directed minimum spanning tree algorithm to extract core directed spillover pathways. Results reveal that China’s urban housing price spillover network exhibits a hierarchical architecture with pronounced ripple effects, where eastern coastal cities and the national core city serve as dominant radiation hubs. The East China sub-network occupies a distinctive net spillover position. We identify heterogeneous structural evolution patterns across regional sub-networks: (1) North China evolved from a dispersed multi-centered configuration to a Beijing-dominated single-core structure; (2) East China developed a robust multi-centered architecture anchored by Shanghai; and (3) South China transitioned from a Guangzhou-centered single-core pattern to a tri-polar configuration co-driven by Guangzhou, Shenzhen, and Nanning.

## 1. Introduction

Real estate constitutes a fundamental pillar of the national economy, with urban housing price fluctuations exerting profound influences on macroeconomic dynamics and household consumption behavior through wealth and substitution effects [1]. Since 2003, China’s housing prices have exhibited sustained upward momentum that consistently outpaced GDP growth [2], with a particularly sharp acceleration following the 2008 global financial crisis recovery [3]. These dynamics have intensified spatial spillovers across urban real estate markets, rendering inter-city price linkages a defining structural feature of China’s economic complex system. The 2020 real estate financing regulatory adjustments further exposed latent market credit risks [4], highlighting the urgent need to quantitatively characterize the cross-city spillover transmission mechanisms underlying inter-city housing price correlations. Since urban real estate markets are inherently interconnected rather than isolated economic entities, price shocks originating in one city can propagate regionally and trigger cascading impacts on neighboring markets [5], thereby amplifying the complexity and systemic significance of inter-city housing price correlations. Constructing a rigorous urban housing price complex network therefore serves as an essential analytical prerequisite for quantifying the nonlinear spatiotemporal dependencies and systemic transmission mechanisms between urban nodes.

The existing literature on housing price correlations predominantly follows two methodological trajectories. The first relies on spatial econometric approaches to characterize the spatial dependence structure, spillover mechanisms, and transmission pathways of housing prices. Meen’s seminal work established the “Ripple Effect” theory, proposing that housing price fluctuations originate in economic core cities and diffuse outward in a ripple-like pattern [6]. Brady subsequently validated this spatial diffusion phenomenon for interstate housing prices in the United States using spatial impulse response functions [7]. Extending this line of inquiry to China, Hong et al. employed a bubble autoregressive model with panel data from 35 major cities to empirically document significant spatial dependence in urban housing prices [8], while Zhu et al. advanced a spatiotemporal modeling framework to illuminate the diffusion pathways of Chinese urban housing prices [9]. More recently, network-based approaches have enriched the understanding of inter-city spatial relationships: Chen et al. leveraged migration and investment flow data to uncover hierarchical structures and connectivity patterns in China’s urban network, demonstrating that topological network positions substantially shape cities’ roles in spatial transmission processes [10]. At the cross-country level, Chowdhury et al. applied spatial econometric models to metropolitan areas in the United States, identifying geographic proximity and economic linkages as primary channels driving the spatial propagation of housing price bubbles [11].

The second methodological trajectory relies on time-series models, which have been more extensively applied in the study of housing price spatial spillovers by capturing the dynamic transmission of price signals across cities over time. Representative tools include cointegration tests, Granger causality analysis, vector autoregression (VAR), and their extensions [12,13,14,15]. Early contributions in this strand focused primarily on the sequential geographic diffusion of housing prices. Gupta and Miller employed Granger causality and VAR frameworks to document statistically significant price ripple phenomena across western U.S. cities [16], while Meng et al. drew upon Random Matrix Theory (RMT) to characterize the spatiotemporal dynamics and systemic risk profile of the U.S. housing market, establishing that regime transitions may function as early warning indicators of real estate bubble formation [17]. Lee et al. adopted a Johansen cointegration and error correction model (ECM) to demonstrate that Taipei exercised persistent price leadership over its surrounding regions [18].

As methodological sophistication advanced, scholarly attention shifted toward volatility spillovers and time-varying contagion dynamics. Zeng et al. [19] constructed a GARCH-BEKK framework and documented asymmetric volatility transmission from core to peripheral cities across three major Chinese urban agglomerations. Zheng et al. combined the supremum augmented Dickey–Fuller (SADF) test with a time-varying parameter VAR (TVP-VAR) model, first identifying housing price bubble intervals across 70 cities and subsequently revealing a pronounced intensification of contagion from first-tier to lower-tier cities after 2010, with regulatory interventions materially reshaping transmission pathways [20]. Lu et al. integrated DCC-GARCH with rolling Granger causality tests, finding that spillover intensity exhibited procyclical co-movement with economic cycles, intensifying during expansionary phases and attenuating during periods of regulatory tightening [21].

While these time-series approaches capture richer dynamic interdependencies than spatial econometric models, they face notable methodological constraints. Housing price data are typically recorded at annual, quarterly, or monthly frequencies, yielding sample sizes that rarely exceed 200 observations [16,18,19,20,21]; under such finite-sample conditions, temporal correlation estimates are susceptible to non-negligible statistical bias. Furthermore, spatial autocorrelation inherent in urban data can systematically distort temporal correlation estimates, as demonstrated by Xiao and Gong in the context of urban scaling analysis [22]. Taken together, these constraints underscore the necessity of developing analytical frameworks specifically tailored to finite-length, spatially dependent sequences.

Despite the substantial progress achieved in the housing price spillover literature, three key gaps remain. First, the spatiotemporal fusion characteristics of inter-city housing price dependencies have rarely been jointly incorporated into network construction, with most studies treating spatial and temporal dimensions in isolation. Second, the finite-sample bias problem, arising from time series typically containing fewer than 200 observations, has received insufficient methodological attention in correlation estimation. Third, existing spatial correlation networks commonly suffer from noise contamination: as the number of city nodes expands, the proliferation of inter-city connections grows combinatorially, producing overly dense topologies that obscure core transmission channels and systemically important nodes.

To address these limitations, we develop a nonlinear spatiotemporal integrated model capable of simultaneously capturing the nonlinear spatiotemporal dependencies inherent in inter-city housing price spillovers. The sample is expanded to 296 cities nationwide to enhance the representativeness and generalizability of the findings, enabling a comprehensive mapping of China’s urban housing price spillover network. The directed minimum spanning tree (DMST) algorithm is further employed to filter spurious correlations and extract the backbone spillover structure, upon which systematic analyses of both interregional and intraregional dynamic evolutionary patterns are conducted. The principal contributions of this study are threefold: (1) the CBEDTE method is introduced to improve the accuracy of causal identification in finite-length sequences, mitigating the estimation biases inherent in conventional transfer entropy approaches; (2) CBEDTE is integrated with a spatial gravity model to yield the CBEDTE-GM framework, a novel nonlinear spatiotemporal integrated model purpose-built for investigating housing price spatial spillovers; and (3) the DMST algorithm is deployed to isolate critical spillover pathways while suppressing noise-induced spurious linkages. Collectively, these contributions provide robust empirical foundations for designing differentiated real estate regulatory policies and enable policymakers to precisely identify systemically important cities and preemptively mitigate cross-regional contagion risks.

The remainder of this paper is organized as follows. Section 2 details the methodological framework. Section 3 describes the data sources and presents descriptive statistics. Section 4 reports and discusses the empirical results. Section 5 offers concluding remarks and outlines directions for future research.

## 2. Methodology

Understanding how housing price shocks propagate across cities requires an analytical framework that simultaneously satisfies three substantive demands. First, inter-city housing price relationships are inherently nonlinear: price signals do not diffuse uniformly but are subject to threshold effects, asymmetric responses, and regime-dependent transmission intensities that linear models cannot adequately capture. Second, housing price datasets are fundamentally finite-length sequences, typically containing fewer than 200 annual or quarterly observations, which renders standard correlation and causality estimators susceptible to non-negligible small-sample bias. Third, housing price spillovers are spatiotemporally integrated phenomena: the intensity with which a price shock in one city affects another depends jointly on the temporal lead-lag structure of price dynamics and on geographic and economic proximity between cities. Treating these two dimensions in isolation, as most existing studies do, necessarily yields an incomplete characterization of the transmission mechanism. The methodological framework developed in this section is designed to address all three demands in a unified manner. Specifically, the Correlation-Dependent Balanced Estimation of Diffusion Transfer Entropy (CBEDTE) is introduced to capture nonlinear directional information flow between city-level price series under finite-sample conditions; CBEDTE is subsequently embedded within a spatial gravity model (GM) to incorporate geographic and economic distance as structural determinants of spillover intensity, yielding the CBEDTE-GM integrated framework to identify housing price spillovers.

Transfer entropy (TE), a non-parametric measure rooted in information theory, offers distinctive methodological strengths for investigating housing price spillover effects [23]. Relative to conventional Granger causality tests and variance decomposition techniques, transfer entropy provides a more flexible analytical lens by accommodating nonlinear dependencies and asymmetric information flow characteristics inherent in housing price series [24,25,26].

Three substantive advantages render TE particularly well suited for this research context. First, the method imposes no assumptions regarding functional form and can accommodate non-normal distributions and heavy-tailed empirical characteristics commonly observed in housing price data. Second, TE explicitly quantifies the directionality of information transmission, such that TE(X→Y)≠TE(Y→X), a property that is indispensable for differentiating spillover-originating cities from spillover-receiving cities [25]. Third, TE is scalable to large multivariate systems without incurring the curse of dimensionality associated with VAR-type models, rendering it especially appropriate for the construction of multi-city housing price networks [24].

Nevertheless, conventional transfer entropy estimators exhibit non-trivial limitations when applied to finite-length sequences containing fewer than 200 observations [27,28]. In the analysis of housing price data, insufficient sample sizes arise frequently due to the restricted publication frequency of statistical bureaus, constraints on data availability, and the narrow time windows typically required for policy evaluation. Under such small-sample conditions, standard transfer entropy estimates are susceptible to substantial bias and instability, which may generate spurious causal inferences or cause genuine spillover channels to go undetected. This technical limitation has measurably constrained the deployment of transfer entropy in dynamic network monitoring and real-time early warning systems for housing markets.

To overcome this methodological challenge, we adopt the Correlation-Dependent Balanced Estimation of Diffusion Transfer Entropy (CBEDTE), a correlation-based estimation framework specifically engineered for finite-length sequence analysis. By reducing the estimation bias of transfer entropy across adjacent segments of high-dimensional finite-length sequences, CBEDTE achieves marked improvements in computational accuracy relative to conventional estimators under data-scarce conditions. As a principled extension of the transfer entropy paradigm, CBEDTE quantifies the directional influence that dynamic changes in one system exert upon another by rigorously characterizing information flow and nonlinear variable interactions. This capacity to represent nonlinear dependencies is particularly valuable for elucidating spillover transmission mechanisms in complex systems and for identifying variables that may assume critical nodal roles under specific market conditions. Grounded in this framework, CBEDTE is employed in the present study to construct a housing price spillover network from finite-length time series data, with the objective of furnishing more reliable technical foundations for systemic risk monitoring in urban real estate markets. Nevertheless, a methodological caveat merits explicit acknowledgement before the formal exposition. Although transfer entropy is asymmetric by construction and conditions on the target variable’s own past history, thereby providing a directional, model-free, and distribution-free measure of information flow that is superior to linear Granger causality in accommodating nonlinear dependencies, it does not constitute strict proof of structural causality in the econometric sense. In particular, common macro-level drivers such as nationwide monetary policy cycles, synchronized regulatory interventions (e.g., the 2016 “housing is for living, not for speculation” directive), or aggregate economic fluctuations may generate correlated price co-movements across cities that are observationally difficult to distinguish from genuine bilateral spillover transmission under standard transfer entropy estimation. This concern is not unique to information-theoretic approaches. As Fang [29] and Quan [30] note in the context of gravity-model-based and panel Logit network approaches respectively, observed pairwise co-movements in Chinese city-level housing prices may partly reflect common factor exposure rather than substantive bilateral spillover channels. The directed minimum spanning tree (DMST) filtering procedure adopted in this study partially addresses this concern by retaining only the strongest directed associations in a parsimonious backbone network, thereby suppressing weak linkages that are more plausibly attributable to noise or common-factor co-movement. Nonetheless, the possibility that residual common-factor contamination influences the estimated transfer entropy values cannot be entirely ruled out, and the directional relationships identified in this study should be interpreted as measures of predictive information flow rather than as structural causal mechanisms in a strict sense.

### 2.1. Correlation-Dependent Balanced Estimation of Diffusion Transfer Entropy (CBEDTE)

In the context of housing price spillover analysis, CBEDTE serves as the core causal identification tool for the following reason. Transfer entropy is a model-free and distribution-free measure that directly quantifies the reduction in uncertainty about the future state of a target city’s housing price attributable to the past states of a source city, without imposing any parametric restrictions on the functional form of their relationship. Crucially, transfer entropy is asymmetric by construction, meaning $TE_{Y\to X}\neq TE_{X\to Y}$, which is indispensable for distinguishing spillover-originating cities from spillover-receiving cities in a directed network. However, when applied to the short annual or quarterly housing price series typically available in China, standard transfer entropy estimators incur substantial finite-sample bias. CBEDTE directly addresses this limitation by replacing each entropy term in the standard transfer entropy expression with the Correlation-Dependent Balanced Estimation of Diffusion Entropy (CBEDE) estimator, which has been shown to markedly reduce systematic bias in finite-length sequence analysis. This makes CBEDTE particularly well suited for constructing reliable directed spillover networks from the kind of limited-observation housing price datasets analyzed in this study. To verify the actual advantages of CBEDTE in this paper, the empirical comparison analysis between CBEDTE and traditional transfer entropy (TE) is provided in Appendix C.

**Figure 1:**
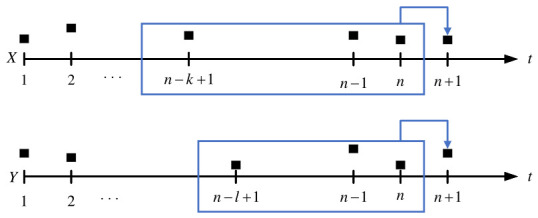
Schematic representation of entropy transfer from sequence Y to sequence X.

For sequence *X*, the conditional probability over all states preceding the n + 1-th state is equivalent to the conditional probability given the *k* states immediately preceding the n + 1-th state. For sequence *Y*, the n + 1-th state is assumed to depend on the preceding *l* states. This dependence structure is illustrated schematically in Figure 1.

In Figure 1, the rectangular blocks correspond to $X_{i}^{\left(k\right)}=\left(X_{i},X_{i-1},\ldots ,X_{i-k+1}\right)$ and $Y_{i}^{\left(l\right)}=\left(Y_{i},Y_{i-1},\ldots ,Y_{i-l-1}\right)$. The transfer entropy from sequence *Y* to sequence *X* is formally expressed as:

(1) $$\begin{array}{c} \begin{array}{c} \;TE_{Y\to X}\left(k,l\right)\\ =H\left(X_{i+1}|X_{i}^{\left(k\right)}\right)-H\left(X_{i+1}|X_{i}^{\left(k\right)},Y_{i}^{\left(l\right)}\right)\\ =\sum p\left(x_{i+1},x_{i}^{\left(k\right)},y_{i}^{\left(l\right)}\right)\log\frac{p(x_{i+1}|x_{i}^{\left(k\right)},y_{i}^{\left(l\right)})}{p(x_{i+1}|x_{i}^{\left(k\right)})}\\ =\sum_{i_{n+1},i_{n}^{\left(k\right)},j_{n}^{\left(l\right)}}p\left(i_{n+1},i_{n}^{\left(k\right)},j_{n}^{\left(l\right)}\right)\log\frac{p\left(i_{n+1},i_{n}^{\left(k\right)},j_{n}^{\left(l\right)}\right)p\left(i_{n}^{\left(k\right)}\right)}{p\left(i_{n+1},i_{n}^{\left(k\right)}\right)p\left(i_{n}^{\left(k\right)},j_{n}^{\left(l\right)}\right)}, \end{array} \end{array}$$

where H(·) denotes the Shannon entropy function; $H\;\left(X_{i+1}|X_{i}^{\left(k\right)}\right)$ is the conditional entropy of $X_{i+1}$ given the *k* most recent preceding states of sequence *X*; and $H\;\left(X_{i+1}|X_{i}^{\left(k\right)},Y_{i}^{\left(l\right)}\right)$ is the conditional entropy of $X_{i+1}$ given both the *k* preceding states of *X* and the *l* preceding states of *Y*, $i_{n}$ denotes the *n*-th element of sequence *X*, and $j_{n}$ denotes the *n*-th element of sequence *Y*. Following standard practice, the order parameters are set to k=l=1 to reduce computational complexity, yielding the simplified expression:

(2) $$\begin{array}{c} TE_{Y\to X}=\sum_{i_{n+1},i_{n},j_{n}}p\left(i_{n+1},i_{n},j_{n}\right)\log\frac{p\left(i_{n+1},i_{n},j_{n}\right)p\left(i_{n}\right)}{p\left(i_{n+1},i_{n}\right)p\left(i_{n},j_{n}\right)}. \end{array}$$

Introducing the substitution $A\equiv i_{n+1}$, $B\equiv i_{n}$, and $C\equiv j_{n}$, Equation (2) can be rewritten as:

(3) $$\begin{array}{c} \begin{array}{c} TE_{Y\to X}=\sum p\left(ABC\right)\log\frac{p(ABC)p(B)}{p(AB)p(BC)}\\ =\sum p(ABC)\log p(ABC)+\sum p(B)\log p(B)\\ -\sum p(AB)\log p(AB)-\sum p(BC)\log p(BC). \end{array} \end{array}$$

Owing to the substantial estimation error inherent in traditional transfer entropy when applied to finite-length sequences, we adopt the Correlation-Dependent Balanced Estimation of Diffusion Entropy (CBEDE) [31]. The mathematical expression for CBEDE is given as follows:

(4) $$\begin{array}{c} {\hat{S}}_{CBEDE}=\frac{1}{L+M}\sum_{j=1}^{M}\left(m_{j}+1\right)\sum_{K=m_{j}+2}^{L+M}\frac{1}{K}, \end{array}$$

In Equation (4), *L* denotes the total length of the sequence, *M* denotes the total number of non-overlapping partitions, referred to as boxes, into which the value range of the original sequence is divided, and $m_{j}$ represents the number of data points, or particles, allocated to the *j*-th box, and *K* is the summation index running from $m_{j}+2$ to L+M, which arises from the combinatorial counting framework underlying the diffusion entropy estimation procedure. To illustrate, consider a sequence whose values lie within [−0.1, 1.1]; setting M=2 partitions this range into the intervals [−0.1, 0.5) and [0.5, 1.1], each constituting an independent box. Tallying the number of observations that fall within each interval enables a more tractable characterization of the distributional properties of the sequence, thereby establishing a systematic basis for subsequent network analysis.

Substituting the CBEDE estimator for each of the four entropy terms in Equation (3) substantially reduces the systematic bias associated with finite-length sequences and yields more stable estimates under data-scarce conditions, thereby enhancing both the accuracy and the robustness of the analysis. The resulting CBEDTE estimator takes the following form:

(5) $$\begin{array}{c} \begin{array}{c} CBEDTE_{Y\to X}=S_{CBEDE}^{ABC}+S_{CBEDE}^{B}-S_{CBEDE}^{AB}-S_{CBEDE}^{BC}\\ \;=\frac{1}{L+M_{ABC}}\sum_{j=1}^{M_{ABC}}(m_{j}^{ABC}+1)\sum_{k=m_{j}^{ABC}+2}^{L+M_{ABC}}\frac{1}{K}\\ \;+\frac{1}{L+M_{B}}\sum_{j=1}^{M_{B}}(m_{j}^{B}+1)\sum_{k=m_{j}^{B}+2}^{L+M_{B}}\frac{1}{K}\\ \;-\frac{1}{L+M_{AB}}\sum_{j=1}^{M_{AB}}(m_{j}^{AB}+1)\sum_{k=m_{j}^{AB}+2}^{L+M_{AB}}\frac{1}{K}\\ \;-\frac{1}{L+M_{BC}}\sum_{j=1}^{M_{BC}}(m_{j}^{BC}+1)\sum_{k=m_{j}^{BC}+2}^{L+M_{BC}}\frac{1}{K}, \end{array} \end{array}$$

In Equation (5), *L* denotes the sequence length and *M* is the number of boxes, conventionally set to 2, which implies $M_{ABC}=2^{3}$ and $M_{AB}=2^{2}$. The quantity $m_{j}^{AB}$ records the number of particles occupying each of the four joint states formed by the binary partitioning of events *A* and *B*.

### 2.2. Modified Gravity Model—CBEDTE-GM

The integration of CBEDTE into the gravity model is motivated by the observation that housing price spillovers between cities are shaped not only by the temporal dynamics of price series but also by structural geographic and economic factors. Empirical evidence consistently demonstrates that spillover intensity decays with physical distance and amplifies with economic mass, patterns that are naturally encoded in the gravity framework [32]. By using CBEDTE as the directional weight in the gravity model, the CBEDTE-GM framework ensures that the resulting spillover intensity matrix $CG_{ij}$ simultaneously reflects: (1) the nonlinear causal influence that city *i*’s housing price dynamics exert on city *j*, as captured by $CBEDTE_{ij}$; (2) the relative economic mass of the two cities, proxied by GDP, registered population, and average housing price level; and (3) the geographic separation between them, measured by the latitude and longitude of the city. This integrated specification thus produces a directed, weighted spillover intensity matrix that is grounded in both the information-theoretic structure of price dynamics and the spatial-economic architecture of China’s urban system, providing a more complete and realistic characterization of inter-city housing price contagion than either component model could achieve in isolation.

The gravity model (GM) has found broad application across diverse domains of urban network analysis, encompassing urban spatial interaction [33,34], population migration [35], transportation flows [36], commercial linkages [37], and environmental and ecological research [38]. Its particular strength lies in quantifying the intensity of cross-regional economic interactions [13,39], which provides a principled foundation for estimating the magnitude of housing price spillovers between cities. Drawing upon prior refinements of the original GM [40], the present study integrates CBEDTE into the gravity model framework to yield the modified CBEDTE-GM model. Three core variables are adopted as “mass” indicators: the average second-hand housing price, regional GDP, and registered population. The distance between cities calculated based on longitude and latitude serves as the “separation” indicator. Together, these variables are incorporated into the CBEDTE-GM model to construct a spatial spillover intensity matrix of housing prices, enabling the quantification of aggregate housing price spillover levels across urban areas nationwide. The selection of the three mass indicators and the functional form of the gravity specification warrant explicit justification. The present model inherits and extends a gravity framework that has been validated in the Chinese urban housing price literature. Fang and Pei applied a closely related specification incorporating housing price, GDP, and population as mass indicators together with a composite distance measure combining geographic distance and per-capita GDP differences, to construct a directed spatial correlation network for 35 major Chinese cities, demonstrating that the resulting network reliably captures hierarchical spillover structures and block-model clustering patterns consistent with economic geography [29]. Quan et al. adopted a comparable gravity specification and further cross-validated it against a panel Logit model, confirming that the gravity-based network structure is robust and that its implied pairwise influence relationships align with econometric estimates [30]. Building on this established foundation, the present study retains housing price $P_{i}$ as the primary mass indicator because it directly measures the market valuation level of city *i* and reflects the magnitude of potential spillover signals; GDP $G_{i}$ proxies the aggregate economic mass that governs both housing demand intensity and the strength of cross-city economic interaction; and registered population $R_{i}$ captures the demographic scale that structurally determines housing demand. The cubic-root aggregation of these three mass indicators follows the specification of Fang and Pei’s study [29], which has been shown to yield a balanced composite measure that avoids dominance by any single indicator. The inverse-square distance decay reflects the well-documented attenuation of spatial economic interaction with geographic separation [39]. The asymmetric directional weight $a_{ij}=P_{i}/\left(P_{i}+P_{j}\right)$ scales spillover intensity by the relative price level of the source city relative to the recipient city, operationalizing the empirical regularity consistently observed in Chinese housing price network studies [29,30], namely that higher-priced markets tend to exert disproportionately stronger outward spillover influence on lower-priced counterparts. Each matrix element $CG_{ij}$ captures the directional spillover intensity transmitted from city *i* to city *j* and can be directly utilized in subsequent spatial network analysis. The CBEDTE-GM model is specified as follows:

(6) $$CG_{ij}=CBEDTE_{ij}\times a_{ij}\frac{\sqrt[{3}]{P_{i}R_{i}G_{i}}\sqrt[{3}]{P_{j}R_{j}G_{j}}}{D_{ij}^{2}},\;a_{ij}=\frac{P_{i}}{P_{i}+P_{j}},$$

where $P_{i}$ and $P_{j}$ denote the housing prices of cities *i* and *j* respectively, $R_{i}$ and $R_{j}$ denote the year-end registered populations, $G_{i}$ and $G_{j}$ denote the respective GDP values, and $D_{ij}$ represents the shortest geographic distance between the two cities.

### 2.3. Directed Minimum Spanning Tree (DMST)

The spatial spillover intensity matrix produced by the CBEDTE-GM model defines a complete directed weighted graph DG=(V,E,CG), in which *V* is the set of *n* city nodes, *E* is the set of directed edges, and $CG=\{CG_{ij}\}$ is the collection of edge weights, where each $CG_{ij}$ quantifies the housing price spillover intensity from city *i* to city *j*. The complete graph comprises n(n−1) directed edges in total. As the number of nodes grows, the resulting network topology becomes prohibitively complex, and the proliferation of weakly associated edges introduces substantial noise that obscures the principal spillover transmission channels and key structural nodes. To resolve this issue, the Directed Minimum Spanning Tree (DMST) algorithm is employed to extract the backbone structure of the housing price spatial spillover network. Minimum spanning tree methods have been successfully applied in financial network analysis [41], international trade network studies [42], and systemic risk contagion research [43], consistently demonstrating their effectiveness in revealing core association patterns within complex systems.

The Edmonds algorithm is adopted to construct the DMST, with the objective of isolating the strongest pairwise spillover associations among cities. Since the minimum spanning tree algorithm selects edges of minimal weight, the spillover intensity values must first be transformed into a distance metric. The normalized spillover intensity matrix satisfies $w_{ij}\in \left[0,\;1\right]$, and the corresponding distance function is defined as:

(7) $$d_{ij}=\sqrt{2\times (1-w_{ij})}.$$

This monotone transformation guarantees that city pairs exhibiting stronger spillover linkages are assigned smaller pairwise distances, ensuring consistency between spillover intensity and edge selection criteria.

The Edmonds algorithm operates on the following principle: it seeks a minimum-weight spanning arborescence rooted at a designated node in a directed graph, with all edges oriented away from the root. The procedural steps are as follows:

1. A directed weighted graph is constructed using the distance matrix $D_{\mathrm{weighted}}=\left\{d_{ij}\right\}$, where each directed edge (i,j) is assigned weight $d_{ij}$.
2. The algorithm automatically identifies the optimal root node and iteratively contracts directed cycles to construct a minimum spanning arborescence comprising exactly n−1 directed edges.
3. Edge directions in the resulting spanning tree are determined endogenously by the algorithm, directly encoding the spillover pathways from source cities to recipient cities.

Relative to conventional undirected minimum spanning tree approaches, the DMST explicitly preserves the directional information embedded in the spillover relationships, thereby enabling a more faithful characterization of the spatial transmission mechanisms underlying housing price dynamics. The final DMST retains only the n−1 strongest directed association edges, effectively suppressing redundant linkages and rendering the core structure of the housing price spatial spillover network clearly identifiable.

### 2.4. Network Evaluation Indicators

To systematically quantify both the aggregate spillover intensity and the systemic importance of individual nodes within the housing price spillover network, a five-dimensional evaluation framework is constructed, comprising the total spillover index, out-degree centrality, standardized betweenness centrality, reverse PageRank index, and composite influence index [44]. Each indicator characterizes the network structure and nodal importance from a distinct analytical perspective: aggregate network spillover intensity, direct radiation capacity of spillover-originating nodes, path intermediation role, cascade propagation potential, and comprehensive hub positioning. Joint examination of these indicators enables multi-dimensional identification of key nodes in the housing price spillover network, furnishing a theoretical basis for regional spillover monitoring, early warning, and differentiated regulatory intervention.

#### 2.4.1. Total Spillover Index

The total spillover index aggregates the pairwise spillover intensities across all city dyads in the network and is formally defined as:

(8) $$TSI\left(t\right)=\sum_{i=1}^{296}\sum_{j=1}^{296}CG_{ij},$$

where $CG_{ij}\left(t\right)$ denotes the gravity-based spillover value transmitted from city *i* to city *j* in year *t*, and TSI(t) represents the nationwide aggregate housing price spillover intensity in year *t*.

#### 2.4.2. Out-Degree Centrality

Out-degree centrality captures the direct transmission capacity of a node in its role as a housing price spillover originator, measured by the number of directed edges emanating from that node toward other nodes in the network. Within the housing price spatial spillover network, this metric reflects the breadth of cities to which a given city’s price fluctuations can directly propagate: a larger value signifies that the city’s housing price movements exert more extensive direct influence across the network. The formal expression is:

(9) $$C_{out}\left(i\right)=\sum_{j=1}^{n}a_{ij},$$

where $a_{ij}$ is an entry of the adjacency matrix, taking the value of 1 if a directed edge exists from city *i* to city *j*, and 0 otherwise; *N* denotes the total number of city nodes in the network. Cities with elevated out-degree centrality typically function as primary initiators of housing price spatial spillovers, with their price fluctuations more readily generating diffusion effects across regional markets. This indicator has been extensively adopted in the analysis of financial risk contagion networks [45].

#### 2.4.3. Standardized Betweenness Centrality

Betweenness centrality quantifies the extent to which a node occupies an intermediary position in spillover transmission, measured as the fraction of shortest paths between all node pairs that pass through the node of interest. In the housing price spatial spillover network, nodes with high betweenness centrality are positioned along critical transmission corridors and may function as structural bridges connecting distinct city clusters or regional sub-networks. To remove the confounding influence of network scale, the standardized formulation is adopted:

(10) $$C_{B}\left(i\right)=\frac{1}{\left(n-1)(n-2\right)}\sum_{s\neq i\neq t}\frac{\sigma_{st}\left(i\right)}{\sigma_{st}},$$

where $\sigma_{st}$ is the total count of shortest paths from city *s* to city *t*, $\sigma_{st}\left(i\right)$ is the number of those paths that pass through city *i*, and *n* is the total number of nodes. Cities with high standardized betweenness centrality act as pivotal relay nodes in the housing price spillover network; pronounced price fluctuations in such cities have the potential to accelerate or amplify inter-regional spillover transmission.

#### 2.4.4. Reverse PageRank Index

While conventional PageRank aggregates influence contributions via incoming edges, the reverse PageRank index performs iterative propagation along outgoing edge directions, thereby measuring the cascade diffusion capacity of a node in its capacity as a spillover source. In the housing price spatial spillover network, cities with elevated reverse PageRank scores not only exhibit strong intrinsic spillover capacity but also transmit price signals to other highly influential cities, thereby generating multi-tier contagion chains. The index is computed as:

(11) $$PR_{rev}\left(i\right)=\frac{1-dc}{N}+dc\sum_{j\in N_{out}\left(i\right)}\frac{PR_{rev}\left(j\right)}{C_{in}\left(j\right)},$$

where $d_{c}=0.85$ denotes the damping coefficient, adopting the standard value; $N_{out}\left(i\right)$ is the set of cities reachable via outgoing edges from city *i*, and $C_{in}\left(j\right)$ is the in-degree of city *j*. The reverse PageRank index is particularly suited to identifying upstream cities that serve as systemic triggers within spillover contagion chains.

#### 2.4.5. Composite Influence Index (CII)

The Composite Influence Index (CII) is designed to provide an integrated assessment of nodal contagion capacity in the housing price spillover network by synthesizing multiple topological dimensions. Whereas individual indicators each capture only a partial facet of nodal influence, the CII consolidates three complementary measures, namely out-degree centrality, betweenness centrality, and reverse PageRank, to jointly characterize a node’s direct propagation capacity, structural intermediation role, and overall influence reach. To preclude subjective weighting bias, the entropy weight method is employed to derive objective indicator weights. The CII is computed as:

(12) $$CII_{i}=100\times \left(w_{1}\cdot \frac{C_{out}\left(i\right)}{\max(C_{out})}+w_{2}\cdot \frac{C_{B}\left(i\right)}{\max(C_{B})}+w_{3}\cdot \frac{PR_{rev}\left(i\right)}{\max(PR_{rev})}\right),$$

where $C_{out}\left(i\right)$ reflects the direct conduction capacity of node *i*, quantified as the number of cities whose housing prices are directly influenced by city *i*; $C_{B}\left(i\right)$ captures the intermediation role of node *i* along housing price propagation pathways; $PR_{rev}\left(i\right)$ represents the reverse-network PageRank score of node *i*, measuring its aggregate influence as a contagion originator; and $w_{1}$, $w_{2}$, $w_{3}$ are objectively calibrated weights derived via the entropy weight method.

The entropy weights are derived objectively from the cross-sectional distribution of each indicator across the 296 city nodes. Table 1 reports the computed weights. Betweenness centrality receives the highest weight ($w_{1}=0.4028$) owing to its greater cross-sectional variation across the minimum spanning tree, while out-degree centrality, being more uniformly distributed in arborescence structures, contributes the lowest information entropy differential and thus receives the lowest weight ($w_{3}=0.2423$).

Within the housing price spillover network, cities achieving high CII scores demonstrate simultaneously strong direct propagation capacity, prominent network intermediation positions, and broad systemic influence, thereby qualifying as core hubs of housing price spatial spillover. Relative to any single topological indicator, the CII is better equipped to surface systemically important cities that perform consistently across multiple network dimensions, offering more robust and comprehensive analytical support for spillover monitoring. The effectiveness of analogous composite scoring approaches in pinpointing influential nodes in complex networks has been well established in prior literature [46].

### 2.5. Weighted Jaccard Similarity Index

To assess the temporal stability and structural persistence of interregional housing price spillover networks, the weighted Jaccard similarity index [47] is adopted as a quantitative measure of network structural resemblance across time. In contrast to conventional binary Jaccard indices that account solely for the presence or absence of network edges, the weighted formulation explicitly incorporates edge weight magnitudes, thereby enabling more precise characterization of temporal variations in spatial spillover intensity.

For any two time points $t_{1}$ and $t_{2}$ at which gravity matrices $G^{\left(t_{1}\right)}$ and $G^{\left(t_{2}\right)}$ have been constructed, the weighted Jaccard similarity $J_{w}$ is formally defined as:

(13) $$J_{w}\left(G^{\left(t_{1}\right)},G^{\left(t_{2}\right)}\right)=\frac{\sum_{i,j}\min\left(G_{ij}^{\left(t_{1}\right)},G_{ij}^{\left(t_{2}\right)}\right)}{\sum_{i,j}\max\left(G_{ij}^{\left(t_{1}\right)},G_{ij}^{\left(t_{2}\right)}\right)},$$

where $G_{ij}^{\left(t\right)}$ denotes the housing price spillover intensity transmitted from region *i* to region *j* at time *t*. The index is bounded within [0, 1], with $J_{w}=1$ indicating perfect structural identity between the two temporal networks and $J_{w}=0$ signifying complete structural divergence.

The distinguishing strength of the weighted Jaccard similarity resides in its explicit sensitivity to edge weight information. The numerator, $\sum_{i,j}\min\left(G_{ij}^{\left(t_{1}\right)},G_{ij}^{\left(t_{2}\right)}\right)$, aggregates the conserved connection strengths shared by both networks, taking the smaller weight at each dyad and thus measuring the stable overlapping component of the two network configurations. The denominator, $\sum_{i,j}\max\left(G_{ij}^{\left(t_{1}\right)},G_{ij}^{\left(t_{2}\right)}\right)$, encompasses the combined scale of both networks by retaining the larger weight at each dyad, thereby capturing the full scope of potential connections and their maximum intensities. Consequently, the index jointly reflects the degree of topological structural similarity and the consistency of edge weight distributions across the two time points.

Systematic examination of the distributional properties, temporal trajectories, and clustering patterns of this index across the observation window facilitates the identification of phased structural transitions, the timing of network reconfiguration breakpoints, and the long-run stability level of the housing price spatial spillover network. These findings furnish quantitative empirical grounding for interpreting the dynamic mechanisms governing regional housing price spatial spillovers in China.

## 3. Data and Descriptive Statistics

The empirical analysis draws on annual housing price data spanning 2000 to 2023, systematically retrieved from the Anjuke real estate platform [48]. Specifically, the housing price variable employed throughout this study refers to the average second-hand residential property listing price for each city in each year. First, with respect to the price type, the data represent listing prices, i.e., the asking prices publicly posted by property sellers on the Anjuke platform, rather than realized transaction prices. Listing prices reflect sellers’ valuation expectations and prevailing market sentiment and have been widely adopted in the Chinese real estate literature as reliable and consistent proxies for city-level housing price levels, particularly in large-sample multi-city studies where systematic transaction-price records are not uniformly available across all cities and time periods [32]. Second, with respect to property type, the dataset covers exclusively second-hand (resale) residential properties. New-build primary market properties are not included in the sample. Third, with respect to temporal aggregation, the annual average listing price for each city is computed as the arithmetic mean of the 12 monthly average listing prices within each calendar year, where each monthly figure represents the average of all valid second-hand residential listing prices posted on the platform within the corresponding city-month cell. Sporadic missing monthly observations arising from data coverage gaps in less developed cities are addressed through linear interpolation prior to the computation of annual averages, as noted below. Annual regional GDP and registered population figures were obtained from the China Statistical Yearbook [49].

For the purpose of interregional housing price spillover analysis, China is partitioned into seven geographic divisions following established regional classification criteria [50]. Northeast China encompasses Liaoning, Jilin, and Heilongjiang provinces. North China incorporates Beijing Municipality, Tianjin Municipality, Hebei Province, Shanxi Province, and Inner Mongolia Autonomous Region. East China covers Shanghai Municipality alongside Jiangsu, Zhejiang, Anhui, Fujian, Jiangxi, and Shandong provinces. Central China comprises Henan, Hubei, and Hunan provinces. South China encompasses Guangdong Province, Guangxi Zhuang Autonomous Region, and Hainan Province. Southwest China incorporates Chongqing Municipality together with Sichuan, Guizhou, and Yunnan provinces and the Tibet Autonomous Region. Northwest China spans Shaanxi and Gansu provinces, Qinghai Province, Ningxia Hui Autonomous Region, and Xinjiang Uygur Autonomous Region. Taiwan Province, the Hong Kong Special Administrative Region, and the Macao Special Administrative Region are excluded from the analysis owing to data unavailability.

Table 2 reports the missing value statistics for the three core variables. Overall missing rates are 4.63% for house prices, 3.69% for regional GDP, and 11.59% for registered population. Missing data arise predominantly in two contexts: (i) the early sub-period 2000–2003, during which statistical reporting was incomplete for newly established cities; and (ii) geographically remote cities in Tibet, Xinjiang, and Qinghai, where systematic data collection remained limited throughout parts of the sample. This pattern is consistent with well-documented data limitations in Chinese city-level panel datasets. Missing observations were addressed through two complementary procedures: linear interpolation was applied to interior missing values, while a forward-fill strategy was adopted for cities with missing values at the commencement of the sample period, propagating the first available observation to fill the initial gap. Descriptive statistics for all variables are reported in Table 3. To verify that the main findings are not sensitive to the choice of imputation method, we replicate the analysis using mean imputation and KNN imputation (k=5) as alternative strategies; the resulting spillover networks are reported in Appendix A and confirm the robustness of all key conclusions.

Sporadic missing observations in the dataset are addressed through linear interpolation. Descriptive statistics for all variables are reported in Table 3.

**Figure 2:**
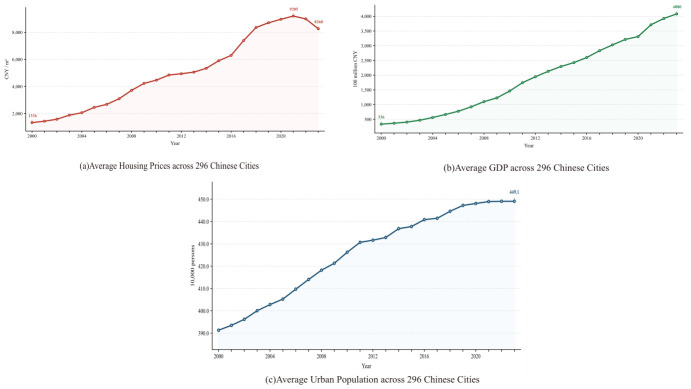
Descriptive Statistics of Key Variables across 296 Chinese Cities, 2000–2023. (**a**) displays the annual mean residential housing price (CNY/m^2^); (**b**) displays the annual mean GDP (100 million CNY); (**c**) displays the annual mean urban population (10,000 persons). All series are averaged across 296 prefecture-level cities for each year.

To further characterize the temporal dynamics of the three core variables, Figure 2 presents the annual cross-city mean trajectories of house prices, regional GDP, and registered population over the 2000–2023 sample period. The average house price exhibits a pronounced and sustained upward trend throughout the observation window, rising from approximately 1336 yuan/m^2^ in 2000 to 8994 yuan/m^2^ in 2023, with the pace of appreciation visibly accelerating after the 2008 global financial crisis recovery. Mean regional GDP displays an equally steep secular increase, reflecting China’s sustained economic expansion over the two decades. By contrast, the mean registered population grows at a comparatively modest rate, rising from approximately 391.6 to 449.1 ten-thousand persons over the observation window—a cumulative increase of only 14.7% across the full 24-year period. Growth was relatively faster during 2000–2010, when urbanization was accelerating, but progressively flattened after 2016, consistent with the deceleration of urbanization and the stabilization of household registration patterns in Chinese cities.

## 4. Results

### 4.1. National Housing Prices Spatial Spillover

#### 4.1.1. National Housing Prices and Spatial Spillover Index Trends

**Figure 3:**
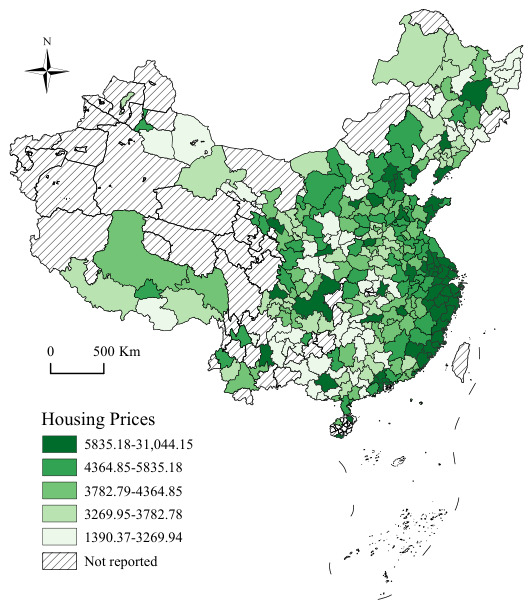
Spatial distribution of average housing prices in Chinese cities (2000–2023 average). Note: The map is produced based on the standard map downloaded from the National Geographic Information Public Service Platform website, with the review number being GS (2024) 0650. The base map has not been modified; the same applies to the following maps.

As depicted in Figure 3, urban housing prices across China exhibit a pronounced spatial stratification pattern, broadly conforming to an east–west gradient in which prices systematically decline from coastal to inland areas. Economically prosperous agglomerations, most notably the Yangtze River Delta and the Pearl River Delta, maintain housing prices substantially above the national average, spanning a range of 5835.18 yuan/m^2^ to 31,044.15 yuan/m^2^ and thereby occupying the apex of the national housing price hierarchy. Other cities along the eastern seaboard, together with selected provincial capitals in central China, constitute an intermediate transitional zone where prices predominantly fall between ¥3782.78 and ¥5835.17 per square meter, tapering progressively as distance from the coast increases. By contrast, the vast western and northern hinterlands are characterized by comparatively subdued price levels, with most cities concentrated within the ¥1390.37 to ¥3782.77 per square meter range. A subset of cities in northwestern and southwestern China are absent from the sample owing to insufficient data coverage.

This geographic price configuration is deeply shaped by the spatial heterogeneity of regional economic development, urbanization intensity, and population concentration. Eastern coastal regions have sustained persistently elevated housing prices by virtue of their geographic advantages, agglomeration economies, and sustained net population inflows. Central and western regions, by contrast, face binding constraints from more moderate economic growth trajectories and comparatively limited population attraction capacity, resulting in structurally lower price levels. The resulting spatial divergence constitutes a vivid manifestation of China’s longstanding uneven regional development landscape.

**Figure 4:**
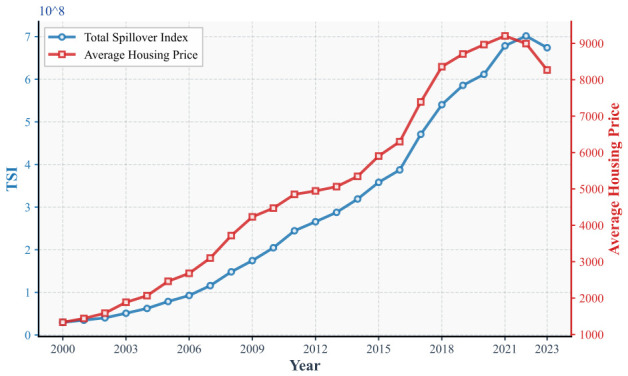
Temporal co-evolution of the national housing price spatial spillover index and average housing price. The red line denotes the average housing price trajectory and the blue line denotes the spillover index trajectory.

Housing price data, population figures, and GDP values are subsequently substituted into Equations (6) and (8) to derive the temporal trajectories of the national housing price spillover index TSI(t) and the average housing price P(t) over the period 2000 to 2023, as presented in Figure 4. Throughout the observation window, TSI(t) expanded from $2.99\;\times \;10^{7}$ to a peak value of $7.02\;\times \;10^{8}$ in 2022, registering a cumulative growth of 2249%. Over the same period, the average housing price P(t) rose from 1336 yuan/m^2^ to 8994 yuan/m^2^, corresponding to a 573% appreciation. Notably, both series recorded their first concurrent decline in 2023, suggesting that housing price spillover dynamics have come under partial restraint. A descriptive examination of Figure 4 reveals that the spillover index series appears to lead the housing price series by approximately one year over portions of the observation window. However, this observation is based solely on visual inspection of trend trajectories and does not constitute formal evidence of a causal lead-lag relationship. A plausible economic interpretation is that market participants require time to perceive, process, and respond to emerging cross-city price signals, thereby introducing a delay between the buildup of network-level spillover pressure and its eventual manifestation in realized housing price levels. Nevertheless, rigorous verification of this lag structure and its underlying transmission mechanism would require dedicated econometric analysis, which we leave as a direction for future research.

#### 4.1.2. National Housing Price Spatial Spillover Network

**Figure 5:**
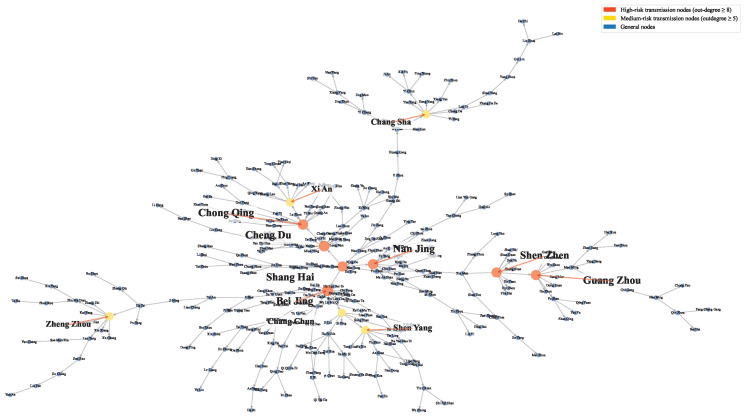
National housing price spatial spillover network. Red nodes identify cities whose direct transmission capacity is greater than or equal to 8, while yellow nodes identify cities whose direct transmission capacity falls within the range of 5 to 7.

To delineate the spatial spillover structure of housing prices across cities and suppress noise embedded in the raw spillover matrix, the CBEDTE-GM model is first applied to construct a spatiotemporal housing price correlation matrix. The directed minimum spanning tree algorithm is subsequently imposed on this matrix to extract the backbone of the national housing price spatial spillover network, as visualized in Figure 5. The resulting network displays a characteristic hierarchical spillover architecture consistent with ripple-effect dynamics, wherein eastern coastal cities and the national capital serve as dominant radiation centers. Price shock propagation follows a discernible stepped gradient, emanating from these core urban nodes and diffusing progressively toward central and western regions.

#### 4.1.3. Topological Analysis of Housing Prices Spatial Spillover Networks

Four topological metrics are jointly employed to characterize the structural properties of the housing price spillover network from complementary analytical perspectives: out-degree centrality, standardized betweenness centrality, reverse PageRank index, and composite influence index. This multi-indicator approach illuminates the heterogeneous roles that individual cities assume within housing price transmission mechanisms.

Out-degree centrality captures the direct transmission reach of each node, quantified as the number of cities to which a given city directly propagates housing price spillovers. As reported in Table 4, Beijing registers the highest out-degree value of 16, positioning it as the foremost housing price spillover initiator in the national network. Chengdu and Chongqing follow with out-degree values of 14 and 10 respectively, consolidating their standing as secondary transmission hubs in the western region. Notably, first-tier cities Shanghai and Shenzhen each record out-degree values of 9, suggesting that their direct spillover reach may be partially attenuated by the dense concentration of economically developed neighboring cities.

Standardized betweenness centrality measures a node’s structural control over network information pathways, operationalized as the share of inter-city shortest paths that traverse the node. Its ranking departs substantially from that of out-degree centrality: Shanghai leads with a coefficient of 0.0116 despite placing only fourth in out-degree, a divergence that underscores its unique intermediation role in bridging eastern coastal cities with inland markets. Fuzhou and Quanzhou rank second and third with coefficients of 0.0089 and 0.0059 respectively, a finding that is particularly striking given the modest economic scale of both cities. This pattern reveals that Fujian Province functions as a pivotal geographic chokepoint in the national housing price spillover network, mediating transmission between the Yangtze River Delta and Pearl River Delta clusters.

The reverse PageRank index quantifies each city’s capacity to initiate cascading housing price shocks, specifically measuring the probability weight that abnormal price movements in a given city will propagate into nationwide spatial spillover effects through iterative backlink propagation. Shanghai records a commanding lead with an index value of 0.09, followed by Fuzhou and Quanzhou. All three cities occupy the spatial intersection of East and South China, collectively constituting a triangular ignition corridor within the national spillover network. The pronounced concordance between this ranking and the standardized betweenness centrality ranking indicates that cities with strong structural bridging capacity simultaneously possess superior spillover triggering potential, a dual functionality that reinforces their centrality within the spatial amplification mechanism.

The Composite Influence Index synthesizes multidimensional topological attributes to produce an integrated assessment of each city’s systemic importance in the housing price spillover network. Shanghai achieves a commanding score of 89.69, affirming its uncontested status as the network’s principal hub. Fuzhou (69.15) and Beijing (62.53) occupy the second and third positions, emerging as the leading secondary nodes. Quanzhou (50.61), Chengdu (45.16), and Shenzhen (43.35) follow in the fourth through sixth positions. Shanghai’s dominance across betweenness centrality, trigger potential, and comprehensive influence simultaneously confirms that it performs a tripartite function encompassing information intermediation, shock initiation, and network aggregation. Fuzhou and Quanzhou, capitalizing on their geographic chokepoint positioning, attain second and fourth place in comprehensive influence, outranking cities of substantially larger economic scale. This outcome highlights the primacy of network topological positioning over raw economic mass in determining a city’s systemic importance. Beijing’s first-place standing in out-degree centrality but third-place position in composite influence further confirms that its network role is predominantly exercised through direct transmission channels rather than through intermediation or cascade initiation.

Integrated examination of all four indicators reveals a stratified hierarchical architecture with three distinct tiers. First, Beijing functions as the dominant direct transmitter, commanding the highest out-degree centrality while ranking third in composite influence. Second, Shanghai achieves pre-eminence across all key dimensions, simultaneously fulfilling intermediation, cascade initiation, and comprehensive influence roles to emerge as the unambiguous core of the spillover network. Third, the Fuzhou–Quanzhou dyad exploits its geographic chokepoint advantage to substantially outperform economically comparable cities in composite influence, securing second and fourth place respectively. This differentiated topological configuration demonstrates that housing price spatial spillovers in China operate through complex multi-pathway mechanisms in which a city’s network prominence is governed by its structural position rather than its economic magnitude alone, thereby validating the multidimensional analytical framework adopted in this study.

#### 4.1.4. Dynamic Ranking of Network Topology Indices

**Figure 6:**
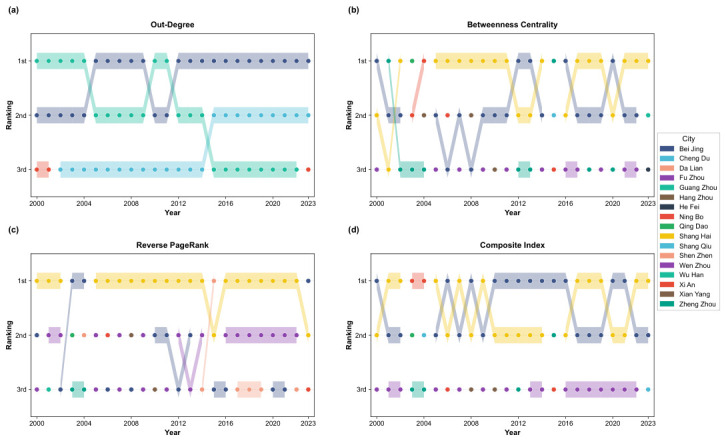
Annual evolution of topological indices in the housing price spatial spillover network. Panels (**a**–**d**) respectively illustrate the temporal evolution of the three highest-ranked nodes each year in terms of (**a**) Out-Degree Centrality, (**b**) Standardized Betweenness Centrality, (**c**) Reverse PageRank Index, and (**d**) Composite Influence Index.

To complement the static network analysis presented in Table 4, the temporal dynamics of topological metrics are examined across the full 2000 to 2023 observation window. Figure 6 traces the annual ranking trajectories of cities across four key centrality measures, uncovering both persistent structural regularities and identifiable transformative phases within China’s housing price spatial spillover network.

Out-degree Centrality (Figure 6a). Beijing retains its leading position throughout the observation window, occupying first place in out-degree centrality for the majority of years, a result fully consistent with its top ranking in Table 4. This sustained dominance attests to Beijing’s durable role as the foremost housing price spillover originator in the national network. Of particular note, Chengdu traces a pronounced ascending trajectory after 2014, advancing to second place and holding this position through 2023, in alignment with its second-place out-degree standing in Table 4. This progression documents Chengdu’s consolidation as a secondary transmission hub within China’s western real estate market.

Standardized Betweenness Centrality (Figure 6b). Shanghai sustains pre-eminent standardized betweenness centrality across the observation period, placing first in the majority of annual rankings. This longitudinal consistency corroborates its highest standardized betweenness centrality score of 0.0116 recorded in Table 4, confirming Shanghai’s indispensable structural role as an intermediary connecting eastern coastal markets with inland counterparts. Fuzhou’s ranking similarly demonstrates remarkable temporal stability, consistently appearing within the top three positions, in line with its second-place entry in Table 4. Despite its comparatively modest out-degree standing, Fuzhou sustains elevated betweenness centrality throughout the sample, underscoring its strategic positioning as a geographic chokepoint regulating housing price transmission between the Yangtze River Delta and Pearl River Delta agglomerations. The persistent divergence between betweenness centrality and out-degree centrality rankings attests to the multidimensional nature of network influence, in which geographic intermediation capacity and direct transmission reach constitute equally indispensable dimensions.

Reverse PageRank Index (Figure 6c). The temporal trajectory of the reverse PageRank index closely mirrors that of betweenness centrality. Shanghai occupies the top position for most years throughout the sample period, consistent with its highest reverse PageRank score of 0.0911 in Table 4, affirming its standing as the principal node in multi-tier contagion chains. Fuzhou recurrently appears among the top three over the majority of the observation window, reinforcing its second-place score of 0.0805 in Table 4. This longitudinal regularity furnishes compelling evidence that cities with strong structural bridging capacity concurrently exhibit greater housing price spatial spillover triggering potential, pointing to the operation of a self-reinforcing feedback mechanism that progressively amplifies systemic network vulnerability.

Composite Influence Index(Figure 6d). Over the 2000 to 2023 period, the composite influence rankings of core network nodes exhibit discernible phased transitions. Beijing and Shanghai rotate between the top-ranked position in composite influence throughout the entire observation period, with their sustained competition defining the structural dynamics at the network apex. After 2012, Fuzhou consistently occupies third place in the composite influence ranking, a pattern that reflects the progressive consolidation of its geographic chokepoint role along the southeastern coastal corridor and the attendant stabilization of its intermediation function within the spillover network. Taken together, the composite influence index discloses a defining characteristic of China’s housing price spillover network: a dual-core dynamic equilibrium prevails at the network center, wherein Beijing and Shanghai sustain joint dominance through cyclical alternation. This observation furnishes a novel analytical perspective for interpreting the long-run evolutionary trajectory of housing price spatial spillovers in China.

**Figure 7:**
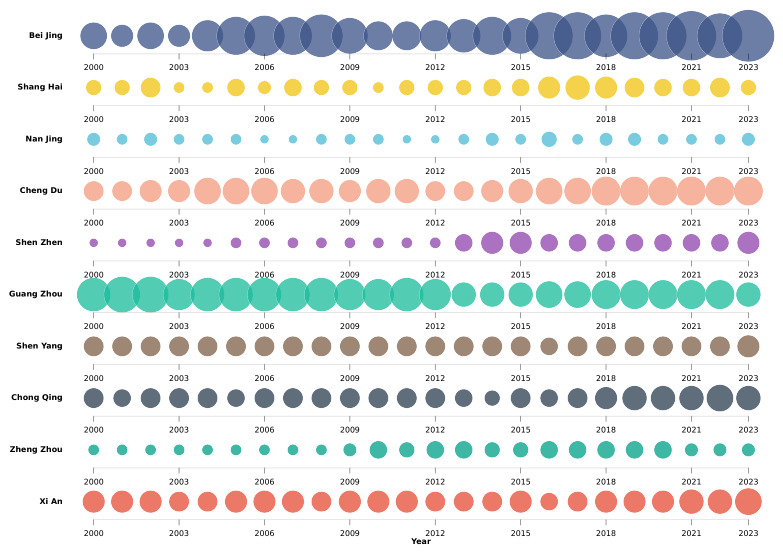
Annual evolution of out-degree centrality among the top ten core cities in the housing price spatial spillover network, 2000–2023. Each row corresponds to one core city; each circle represents one year. Circle size is scaled by the city’s out-degree centrality in the directed minimum spanning tree for that year, reflecting the number of cities to which it directly transmits housing price spillovers. Larger circles indicate broader direct transmission reach in the corresponding year.

Out-Degree Centrality Evolution in Top Ten Cities. Figure 7 presents the annual evolution of out-degree centrality for ten core cities, including Beijing, Shanghai, Guangzhou, and Chengdu, over the period 2000 to 2023. Each circle’s size reflects the city’s out-degree centrality in the directed minimum spanning tree for that year, representing the number of cities to which it directly transmits housing price spillovers. Beijing, Guangzhou, and Chengdu each sustain persistently large out-degree values over the long run, cementing their enduring centrality within the national housing price spatial spillover network as primary direct transmitters. As the principal northern hub, Beijing maintains a consistent lead in out-degree centrality, reflecting its entrenched dominance in directly transmitting housing price signals across North and Northeast China and corroborating its dual status as a political and economic center. Shanghai and Shenzhen exhibit a secular upward trend in out-degree centrality, capturing the progressive broadening of the direct transmission reach of core cities in the Yangtze River Delta and Pearl River Delta regions as economic development deepens and population concentration intensifies. Central and western cities, most notably Chengdu and Xi’an, sustain broadly stable out-degree values throughout the sample, reflecting their established function as regional secondary hubs that maintain consistent direct linkages with surrounding cities, thereby corroborating the hierarchical architecture of housing price spatial spillover.

### 4.2. Interregional Housing Prices Spatial Spillover

#### 4.2.1. Pathways of Interregional Housing Price Spatial Spillover

A 24-year interregional housing price spillover gravity matrix is constructed from annual data spanning 2000 to 2023. To assess the temporal stability of the network structure, pairwise comparisons of annual networks are conducted using the weighted Jaccard similarity index. A 24×24 temporal similarity matrix *S* is computed for each year pair $\left(t_{i},t_{j}\right)$ within the 2000 to 2023 window.

**Figure 8:**
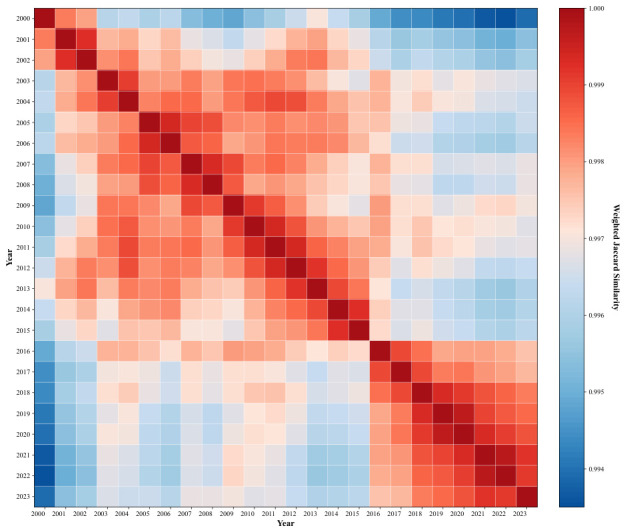
Temporal similarity matrix of interregional housing price spillover networks from 2000 to 2023. Each cell reports the weighted Jaccard similarity between the edge sets of year *t* (y-axis) and year *s* (x-axis); red tones denote high structural similarity in spillover linkages, while blue tones indicate comparatively greater topological divergence.

The pairwise comparison results are presented as a heatmap in Figure 8. While the overall weighted Jaccard similarity coefficients remain consistently high across all inter-period comparisons, a closer inspection of the heatmap reveals a salient two-block diagonal structure that carries substantive economic meaning. Specifically, the heatmap exhibits two distinct high-similarity clusters along the main diagonal, separated by a clearly visible band of comparatively lower similarity (blue-tinted cells), with the structural break occurring at approximately 2015–2016. This two-block pattern is not a statistical artifact but instead encodes two distinct network regimes in China’s interregional housing price spillover architecture, each driven by an identifiable macroeconomic and policy environment.

The first block (2000–2015) corresponds to the prolonged market expansion phase following China’s housing commercialization reform. Throughout this period, urban housing prices exhibited sustained upward momentum, and the interregional spillover network progressively consolidated around eastern coastal cities as dominant radiation hubs. Although notable macroeconomic disturbances occurred within this window, most prominently the 2008 global financial crisis and the subsequent four-trillion-yuan fiscal stimulus package, these shocks accelerated the pace of price appreciation without fundamentally altering the directional architecture of the spillover network. The high intra-block similarity observed across 2000–2015 thus reflects a period of continuous and uninterrupted network evolution operating within a stable structural regime. It is also worth noting that the year 2000 displays a slightly lower similarity with later years within this block, which is attributable to an endpoint effect arising from its position at the commencement of the observation window rather than signaling a genuine structural break.

The second block (2016–2023) is demarcated by a sharp structural discontinuity at the 2015–2016 boundary, visible as a pronounced transition from red to blue tones in the off-diagonal cells connecting the two blocks. This break coincides precisely with the landmark “housing is for living, not for speculation” directive formally established in 2016, which represented a fundamental reorientation of China’s real estate regulatory philosophy. Prior to this intervention, housing markets had operated under a broadly accommodative policy environment that tacitly permitted speculative demand accumulation and price appreciation across city tiers. The 2016 directive decisively reversed this stance, introducing a comprehensive package of demand suppression measures including tightened purchase restrictions, stricter mortgage qualification criteria, and enhanced local government accountability for price stability. These interventions collectively recalibrated price dynamics across the urban hierarchy, compressing speculative transmission channels that had previously amplified cross-city spillovers and thereby inducing a durable reconfiguration of the interregional spillover network. Under this new regulatory regime, the spatial transmission pathways that had characterized the first block were materially disrupted: spillover intensities contracted, the radiation dominance of top-tier cities was partially attenuated, and the network topology converged toward a new structural equilibrium that persisted through 2023. The high intra-block similarity observed within 2016–2023 confirms that this reconfigured network architecture stabilized rapidly following the initial policy shock and remained robust thereafter.

Taken together, the two-block diagonal structure of Figure 8 discloses a fundamental duality in China’s interregional housing price spillover dynamics: a prolonged expansion regime spanning 2000–2015, characterized by progressive network consolidation under market forces, and a policy-induced reconfiguration regime spanning 2016–2023, characterized by structural network adjustment under the tightening regulatory environment inaugurated by the directive. The pronounced between-block dissimilarity relative to within-block cohesion confirms that China’s regional housing price spillover network, while exhibiting considerable structural inertia within each regime, undergoes abrupt and durable reconfiguration at regime boundaries triggered by major policy interventions. These findings carry direct implications for macroprudential policy design: regulatory measures of sufficient magnitude and philosophical clarity do induce network-level structural breaks, and the temporal boundaries of these breaks are clearly legible in the block structure of the temporal similarity matrix.

**Figure 9:**
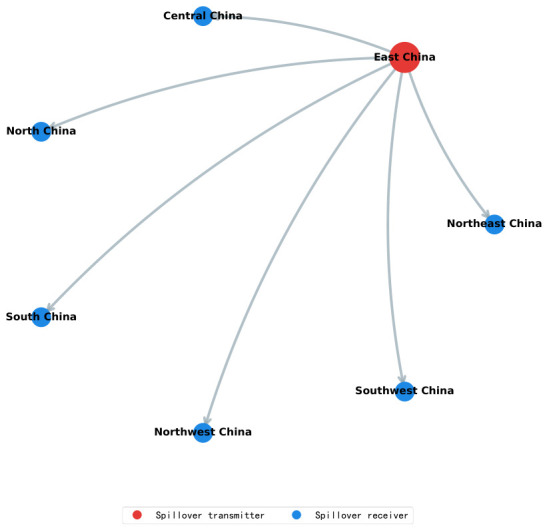
Interregional housing price spatial spillover network structure. Arrows denote the direction of spillover transmission from source regions to recipient regions.

Grounded in this confirmed temporal robustness, the spatial architecture of interregional housing price spillovers is examined further. Figure 9 portrays the structural configuration of spillover pathways across China’s seven major geographic divisions. East China persistently occupies the central hub position in the network, serving as the sole spillover originator and transmitting price signals unidirectionally to North China, Central China, South China, Northeast China, Northwest China, and Southwest China. This architecture conforms to a canonical core-periphery unidirectional radiation pattern. These findings attest to the commanding role of East China, anchored by the Yangtze River Delta urban agglomeration, in the national housing price discovery process, while the remaining regions operate predominantly as passive recipients and downstream responders to incoming price signals.

#### 4.2.2. Housing Price Spatial Spillover from East China to Other Regions

**Figure 10:**
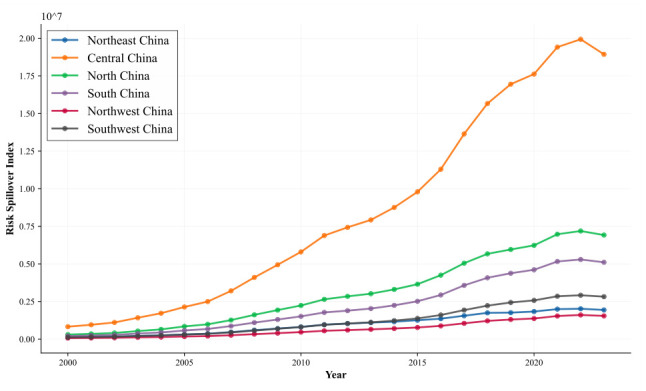
Temporal evolution of housing price spatial spillover from East China to other regions. Each line corresponds to the spillover trajectory directed toward a specific recipient region.

Figure 10 traces the temporal evolution of housing price spatial spillover transmitted from East China to each of the remaining six major regions over 2000 to 2023. The data reveal pronounced spatial heterogeneity in East China’s outward spillover effects, with this heterogeneity becoming more accentuated over time. Central China functions as the predominant recipient throughout the observation window, sustaining the highest absorbed spillover volume across all years. Absorbed spillover in Central China surges from $0.87\;\times \;10^{6}$ in 2000 to $1.87\;\times \;10^{7}$ in 2023, a more than twentyfold expansion that substantially exceeds the growth recorded in all other recipient regions.

North China ranks as the second-largest absorbing region, with its received spillover volume rising from $0.61\;\times \;10^{6}$ in 2000 to $6.85\times 10^{6}$ in 2023, representing a greater than tenfold accumulation. By contrast, the Northeast, Northwest, and Southwest regions register comparatively modest spillover volumes accompanied by more subdued growth trajectories. This spatial stratification reveals that housing price fluctuations originating in East China generate disproportionately stronger radiating effects on geographically adjacent and economically integrated regions, specifically Central and North China, while transmission intensity attenuates markedly with increasing spatial separation. These patterns are consistent with pronounced distance-decay dynamics and economic-linkage-oriented transmission channels.

#### 4.2.3. East China’s Contribution to National Housing Price Spillover

**Figure 11:**
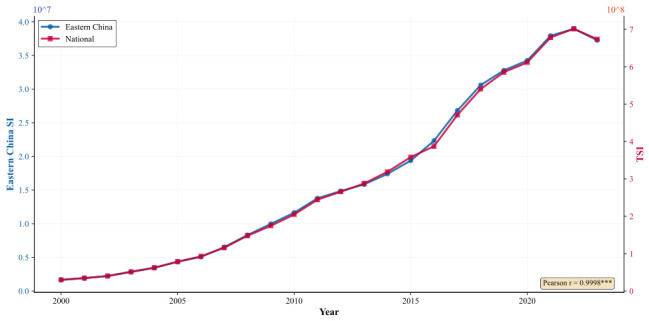
Temporal co-evolution of the total housing price spatial spillover in East China and the national aggregate housing price spatial spillover. The blue solid line traces East China’s total outward spillover, while the red line traces the national total housing price spatial spillover.

To further quantify East China’s role within the national housing price spatial spillover network, Figure 11 presents the temporal co-evolution of East China’s aggregate outward spatial spillover and the national total housing price spillover. The two series track each other with remarkable fidelity throughout the sample period. East China’s total outward spatial spillover expands from $1.66\;\times \;10^{6}$ in 2000 to $3.72\;\times \;10^{7}$ in 2023, while the national total housing price spillover rises from $2.99\;\times \;10^{7}$ to $6.74\;\times \;10^{8}$ over the same interval. The Pearson correlation coefficient between the two series reaches 0.9998 (p<0.001), confirming an exceptionally strong positive co-movement.

In summary, the gravity-model-based regional housing price spatial spillover network of China is characterized by three overarching systemic properties. First, with respect to topological stability, the network sustains a stable core-periphery unidirectional radiation architecture across the entire observation period. East China persistently occupies the sole spillover originator position, while the remaining six major regions continuously serve as downstream spillover recipients. This durable role configuration indicates that China’s regional housing price spatial spillover network possesses considerable structural inertia, rendering it resistant to fundamental reconfiguration even under external shocks.

Second, concerning spatial heterogeneity, East China’s outward spillover exhibits pronounced distance-decay properties and strong economic-linkage orientation. Transmission intensity reaches its apex in geographically adjacent and economically integrated Central and North China, while remaining comparatively subdued in peripheral regions. This spatial stratification discloses the joint operation of geographic proximity forces and economic gravitational pull as the dual drivers of spatial spillover propagation.

Third, the temporal trajectory of East China’s total housing price spatial spillover demonstrates near-perfect synchronization with the national aggregate. Convergent empirical evidence across these three dimensions establishes that East China operates simultaneously as a systemic spillover originator and a dynamic engine of cross-regional housing price diffusion. Its price fluctuations not only shape the topological architecture of the national spatial spillover network but also govern the temporal evolution path of aggregate housing price spillovers nationwide. These findings furnish an essential complex-network perspective for deciphering the macro-level price transmission mechanisms that underpin China’s real estate market.

### 4.3. Housing Price Spatial Spillover Within Regions

#### 4.3.1. Evolution of Housing Price Spatial Spillover Networks in North China

(1) Housing Price Spatial Spillover Network in North China.

**Figure 12:**
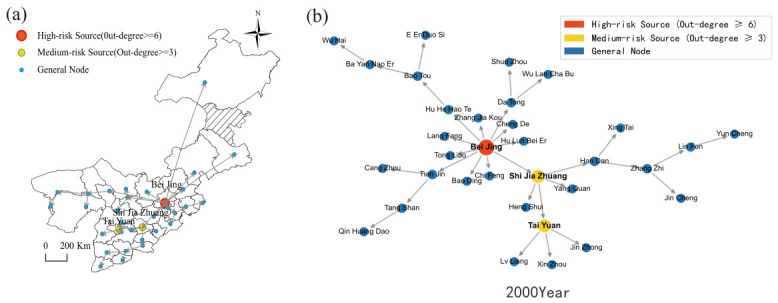
Housing price spatial spillover network in North China in 2000: (**a**) Geographic distribution of nodes in the study region, distinguishing high spillover sources (out-degree ≥ 6, red circles), medium spillover sources (out-degree ≥ 3, yellow circles), and general nodes (blue circles). Scale bar represents 200 km. (**b**) Network topology derived from the DMST algorithm, depicting directional transmission relationships between nodes. Node colors carry the same classification as in panel (**a**).

Figure 12 reveals that the housing price spatial spillover network in North China in 2000 was characterized by a polycentric configuration featuring multiple secondary hubs with dispersed coverage. Although Beijing occupied a prominent nodal position, its structural dominance had not yet been fully consolidated. Shijiazhuang and Taiyuan each functioned as independent secondary transmission hubs, generating direct spatial spillover linkages with their respective neighboring cities. The influence zones of these secondary hubs remained largely non-overlapping, yielding an overall network topology marked by geographically scattered hubs and regionally compartmentalized connections.

**Figure 13:**
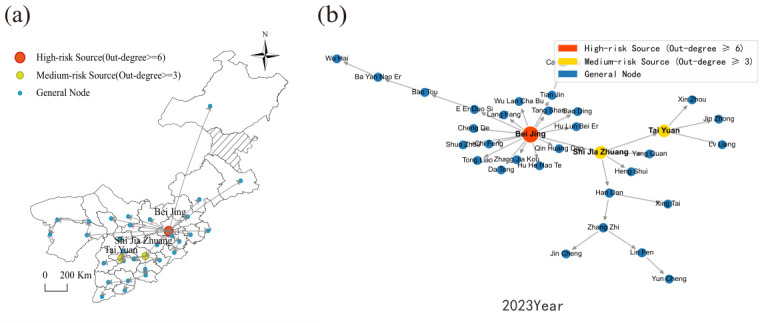
Housing price spatial spillover network in North China in 2023: (**a**) Geographic distribution of nodes in the study region. Node colors reflect spillover intensity levels following the same classification as Figure 12. Scale bar represents 200 km. (**b**) Network topology derived from the DMST algorithm, depicting directional transmission relationships between nodes. Node colors carry the same classification as in panel (**a**).

By 2023, the network had undergone a fundamental structural transition. Figure 13 demonstrates unambiguously that the network converged toward a monocentric architecture centered on Beijing. Assuming the role of the region’s sole dominant hub, Beijing forged dense and far-reaching direct spatial spillover linkages with cities including Tianjin, Langfang, and Baoding. Virtually all inter-city housing price co-movements within North China are now channeled through Beijing as the central intermediary node. The autonomous influence previously wielded by former secondary hubs has contracted substantially, and the network has crystallized into a Beijing-anchored agglomeration structure.

(2) Topology of Housing Price Spatial Spillover Networks in North China Cities.

**Figure 14:**
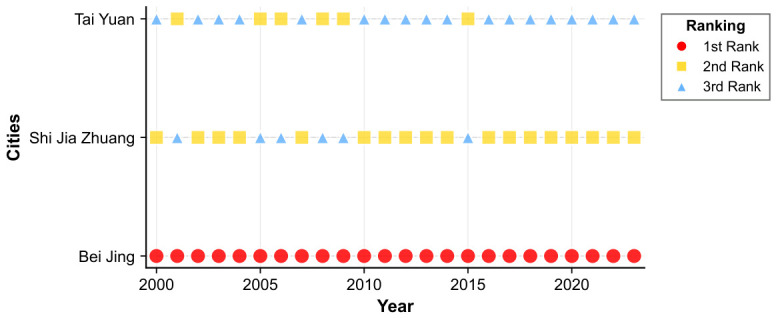
Annual evolution of central cities in the housing price spatial spillover network in North China. Circles denote the city with the highest direct radiation capacity in each year, squares denote the city with the second highest direct radiation capacity, and triangles denote the city with the third highest direct radiation capacity.

Figure 14 documents that Beijing, Shijiazhuang, and Taiyuan consistently constitute the top three cities in North China’s housing price spillover network throughout the observation period. Interpreted alongside the network structural evidence presented above, this ranking confirms that Beijing’s leadership capacity has progressively intensified over time, while the spillover transmission strength of secondary hub cities has undergone a gradual but sustained decline.

#### 4.3.2. Evolution of Housing Price Spatial Spillover Networks in East China

(1) Housing Price Spatial Spillover Network in East China.

**Figure 15:**
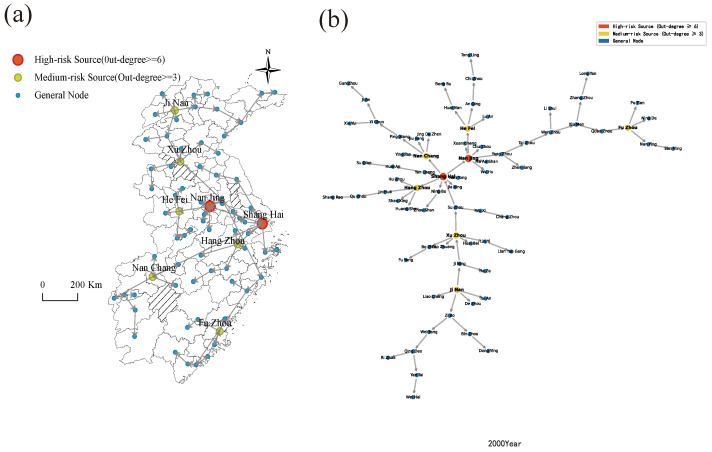
Housing price spatial spillover network in East China in 2000: (**a**) Geographic distribution of nodes in the study region, distinguishing high spillover sources (out-degree ≥ 6, red circles), medium spillover sources (out-degree ≥ 3, yellow circles), and general nodes (blue circles). Scale bar represents 200 km. (**b**) Network topology derived from the DMST algorithm, depicting directional transmission relationships between nodes. Node colors carry the same classification as in panel (**a**).

**Figure 16:**
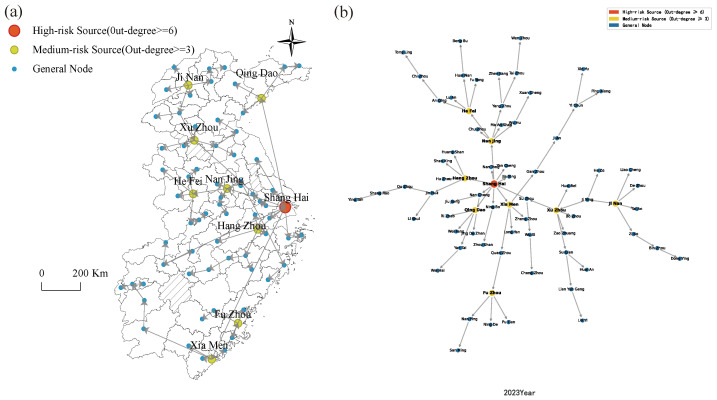
Housing price spatial spillover network in East China in 2023: (**a**) Geographic distribution of nodes in the study region. Node colors reflect spillover intensity levels following the same classification as Figure 15. Scale bar represents 200 km. (**b**) Network topology derived from the DMST algorithm, depicting directional transmission relationships between nodes. Node colors carry the same classification as in panel (**a**).

The housing price spatial spillover network in East China experienced pronounced structural reorganization over the 2000 to 2023 period. Given space limitations, the analysis focuses on the endpoint years of 2000 and 2023 (Figure 15 and Figure 16). Figure 15 documents a canonical core-periphery hierarchical architecture in the 2000 network, wherein housing price spillovers emanated predominantly from Shanghai and Nanjing as the twin primary radiation centers.

By 2023, the network topology had transitioned into a dense, multi-core configuration. As portrayed in Figure 16, the network is anchored by multiple secondary hub nodes operating within an overarching multi-core structure. Shanghai retains its position as the principal spatial spillover originator, channeling price fluctuations to Jiangsu, Zhejiang, and Jiangxi provinces, while provincial capitals across East China serve as secondary hubs that relay price movements directly to cities within their respective provincial jurisdictions.

This multipolar hub architecture effectively distributes price transmission pressures across the network. Idiosyncratic market volatility in any single city is no longer capable of instantaneously propagating systemic shocks throughout the entire region, a property that reflects the enhanced resilience intrinsic to a structurally mature network.

(2) Topological Structure of Housing Price Spatial Spillover Networks in East China Cities.

**Figure 17:**
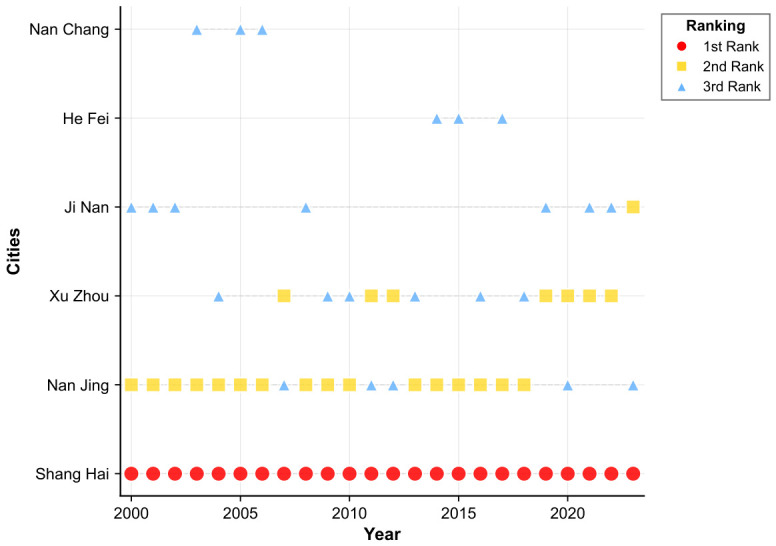
Annual evolution of central cities in the housing price spatial spillover network in East China. Circles denote the city with the highest direct radiation capacity in each year, squares denote the city with the second highest direct radiation capacity, and triangles denote the city with the third highest direct radiation capacity.

Figure 17 traces the annual evolution of central cities within East China’s housing price spatial spillover network over 2000 to 2023. The resulting topology is decentralized in structure yet coherently organized, with Shanghai functioning as the uncontested core around which multiple cities operate in coordinated complementarity. The network exhibits pronounced hierarchical differentiation, constituting a multi-tiered housing price spillover system encompassing the six provinces and one municipality of East China.

Shanghai maintained uninterrupted core positioning throughout 2000 to 2023. Capitalizing on its status as an international financial center, Shanghai deploys its commanding economic strength, financial resource aggregation capacity, and policy signaling effects to anchor its role as the pre-eminent spatial housing price spillover source in East China. Price spillover signals propagate from Shanghai to surrounding provinces along the most efficient transmission pathways, and this centrality persists without interruption across the observation window, attesting to Shanghai’s entrenched leadership within the East China real estate market.

Nanjing consistently occupies the second-ranked position among network central cities for the overwhelming majority of the observation period, assuming the role of a structural second core. As the secondary hub of the Yangtze River Delta and the provincial capital of Jiangsu, Nanjing operates as an indispensable bridge articulating the Shanghai–Nanjing–Hangzhou urban cluster with the Anhui River Urban Belt. Its housing price dynamics simultaneously absorb upstream spillover from Shanghai and radiate downstream to surrounding second-tier and third-tier cities, positioning it as a critical relay fulcrum within the regional network.

Xuzhou commands a distinctive geographic advantage at the convergence of Jiangsu, Shandong, Henan, and Anhui provinces, functioning as a cross-boundary regional hub that bridges East China with North China and links coastal markets with inland counterparts. Its housing price spillover exerts substantial influence over the city cluster of the Huaihai Economic Zone. Provincial capitals including Fuzhou, Hefei, and Jinan intermittently ascend to the top three central city positions, each serving as the dominant spillover node within its respective provincial housing price network.

#### 4.3.3. Evolution of Housing Price Spatial Spillover Networks in South China

(1) Housing Price Spatial Spillover Network in South China.

**Figure 18:**
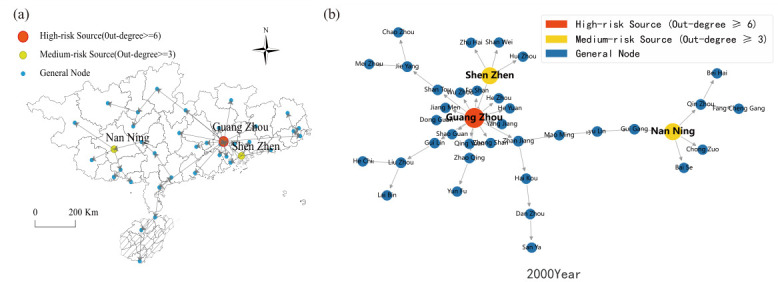
Housing price spatial spillover network in South China in 2000: (**a**) Geographic distribution of nodes in the study region, distinguishing high spillover sources (out-degree ≥ 6, red circles), medium spillover sources (out-degree ≥ 3, yellow circles), and general nodes (blue circles). Scale bar represents 200 km. (**b**) Network topology derived from the DMST algorithm, depicting directional transmission relationships between nodes. Node colors carry the same classification as in panel (**a**).

Figure 18 reveals that the 2000 housing price spatial spillover network in South China was dominated by a monocentric structure anchored on Guangzhou. The majority of cities in the Pearl River Delta and western Guangdong received direct housing price spillover influence emanating from Guangzhou, manifesting a pronounced single-core radiation pattern. Shenzhen retained direct linkages with only a limited number of cities, principally Huizhou and Shanwei, while Nanning maintained comparatively tenuous ties to the South China core cluster. The overall network topology consequently assumed a single-core core-periphery configuration.

**Figure 19:**
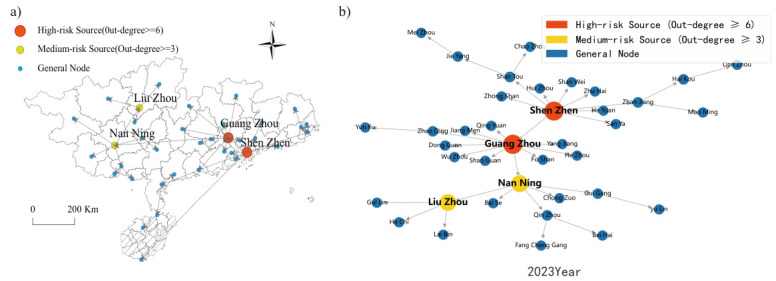
Housing price spatial spillover network in South China in 2023: (**a**) Geographic distribution of nodes in the study region. Node colors reflect spillover intensity levels following the same classification as Figure 18. Scale bar represents 200 km. (**b**) Network topology derived from the DMST algorithm, depicting directional transmission relationships between nodes. Node colors carry the same classification as in panel (**a**).

By 2023, a fundamental structural shift had materialized. As portrayed in Figure 19, the network had reorganized into a tricentric driving configuration anchored by Guangzhou, Shenzhen, and Nanning. Guangzhou retained its position as the central hub, while Shenzhen, serving as the integrating core of eastern Guangdong, substantially extended its radiation reach. Nanning, operating as the western regional core, forged dense spillover linkages with Guangxi cities including Liuzhou. The overall network architecture evolved into a complex multi-centered topology characterized by distributed hub support and regionally differentiated transmission patterns.

(2) Topological Structure of Housing Price Spatial Spillover Networks in South China Cities.

**Figure 20:**
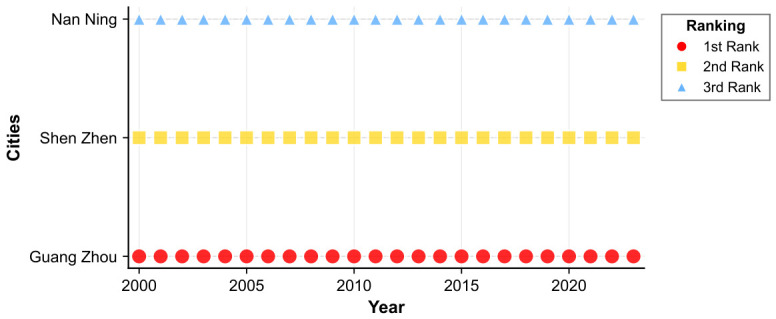
Annual evolution of central cities in the housing price spatial spillover network in South China. Circles denote the city with the highest direct radiation capacity in each year, squares denote the city with the second highest direct radiation capacity, and triangles denote the city with the third highest direct radiation capacity.

Figure 20 traces the annual evolution of central cities within South China’s housing price spatial spillover network. The centrality hierarchy and temporal evolution characteristics of the core cities are detailed as follows.

Guangzhou sustained the highest degree centrality throughout the 2000 to 2023 period, anchoring its position as the principal node of South China’s housing price spillover network and exerting persistent direct influence over cities across the Pearl River Delta, eastern Guangdong, and western Guangdong. Shenzhen followed a steady ascending trajectory over the sample period, progressively transitioning from a secondary node to a co-core node, a trajectory that mirrors the broadening footprint of its real estate market influence across the region.

Nanning registered comparatively subdued centrality in the early 2000s but subsequently underwent rapid elevation in network standing. It ultimately emerged as the spatial spillover core for housing prices across western South China, filling the structural vacuum previously present at the network’s western periphery. Collectively, these three cities underpin the tricentric architecture of South China’s 2023 housing price spillover network, instantiating a layered core framework organized around the Guangzhou–Shenzhen–Nanning triad.

Over the full 2000 to 2023 observation window, South China’s housing price spatial spillover network transitioned from a Guangzhou-dominated monocentric model to a Guangzhou–Shenzhen–Nanning tricentric-driven system. This structural evolution is jointly attributable to Shenzhen’s ascent as an economic and real estate powerhouse within the Pearl River Delta and to Nanning’s consolidation of influence over western South China. Under this tri-core configuration, housing price spillover dynamics exhibit heightened multi-source complexity, necessitating that regional real estate regulatory frameworks strike a calibrated balance between managing core city contagion linkages and implementing differentiated sub-regional policy interventions.

## 5. Conclusions

Inter-city housing price linkages have assumed growing importance for deciphering systemic transmission mechanisms and generating reliable forecasts of regional housing price dynamics. Prevailing research confronts a fundamental methodological tension: spatial econometric approaches effectively characterize geographic spillover mechanisms yet neglect temporal dynamics [51], whereas time-series methods such as Granger causality and VAR models accommodate dynamic transmission [52] but incur substantial estimation biases stemming from limited sample sizes, given that housing price datasets typically contain fewer than 200 observations [16,18,19,20,21,53]. To resolve these compounding challenges, the present study develops a novel nonlinear spatiotemporal integrated model that jointly captures both spatial and temporal dimensions of housing price spillovers. The correlation-dependent balanced estimation of diffusion transfer entropy (CBEDTE) is introduced to sharpen causal identification in finite-length sequences, systematically attenuating the biases inherent in small-sample estimation. CBEDTE is subsequently integrated with a spatial gravity model to yield the CBEDTE-GM framework, which simultaneously accounts for geographic proximity effects and dynamic transmission mechanisms. The directed minimum spanning tree (DMST) algorithm is further deployed to suppress spurious correlations and isolate critical spillover pathways. With sample coverage extended to 296 cities nationwide, the complete spillover network of Chinese urban housing prices is mapped and the dynamic evolutionary patterns of both interregional and intraregional spatial spillover effects are systematically documented.

At the national level, the housing price spatial spillover network exhibits a characteristic ripple-effect hierarchical architecture, with eastern coastal cities and the national capital serving as the dominant radiation nodes. Interregional analysis establishes that East China persistently anchors the network’s core hub position, functioning as the principal spillover originator that continuously transmits price shocks to the remaining six major regions, thereby instantiating a canonical core-periphery unidirectional radiation pattern. Notably, East China’s outward spillover intensity peaks toward Central and North China, a spatial distribution that is consistent with pronounced distance-decay dynamics and strong economic-linkage orientation.

Zooming into intraregional dynamics, the analysis uncovers distinct structural evolutionary trajectories across regional sub-networks. North China underwent a transition from a polycentric configuration with dispersed secondary hubs to a Beijing-anchored monocentric structure. East China consolidated a resilient multi-centered topology with Shanghai as the uncontested core, Nanjing as a strategic relay pivot, and a constellation of other cities operating in coordinated complementarity. South China shifted from a Guangzhou-centered monocentric framework to a tricentric system jointly driven by Guangzhou, Shenzhen, and Nanning. Collectively, these findings illuminate the regional linkage architecture underlying China’s sustained large-scale housing price appreciation and furnish essential complex-network insights for deciphering the systemic transmission mechanisms at work.

The network characteristics identified in this study carry substantive implications for both forecasting and regulatory design. At the node level, the designation of Beijing and Shanghai as systemically important spillover initiators enables more targeted prediction of shock origination points and propagation trajectories. Cities with elevated out-degree centrality serve as leading indicators whose price movements foreshadow forthcoming adjustments in downstream connected cities, while nodes with high betweenness centrality function as critical transmission conduits whose price dynamics yield early warning signals for cross-regional contagion. At the path level, the directional spillover channels documented here, most notably East China’s role as the net spillover source toward Central and North China, expose the upstream–downstream logic of price transmission and enable region-specific forecasting models that explicitly incorporate network directionality. At the structural level, the heterogeneous topologies identified across regions call for differentiated analytical approaches: Beijing’s dominant position in North China’s monocentric network confers strong predictive leverage on the capital’s price movements; East China’s resilient multi-hub structure necessitates concurrent surveillance of multiple central cities; and South China’s evolving tri-core configuration requires joint monitoring of the coordinated price dynamics among Guangzhou, Shenzhen, and Nanning. Taken together, these findings furnish a robust empirical foundation for designing region-differentiated forecasting models and macroprudential regulatory frameworks for China’s real estate market.

Several limitations of the present study warrant acknowledgement and point toward productive avenues for future inquiry. First, while CBEDTE substantially improves estimation performance relative to conventional transfer entropy under finite-sample conditions, its accuracy for sequences containing fewer than 200 observations leaves room for further refinement. Second, the network framework adopted here operates within a single-layer structure; as richer datasets become available, extending the analysis to multi-layer complex network representations could yield more nuanced insights into the simultaneous operation of multiple interdependency channels. Third, the present analysis centers on the topological properties of the housing price spillover network; subsequent work should investigate the transmission mechanisms through which macroeconomic variables and policy interventions shape and reshape these spillover dynamics. Finally, the estimated transfer entropy values may nonetheless be partially confounded by common macroeconomic factors such as synchronized monetary policy cycles or coordinated nationwide regulatory interventions that induce correlated price co-movements across cities independently of genuine bilateral spillover transmission. This challenge is shared by alternative network construction approaches [29,30] and represents a broader methodological frontier in the housing price network literature. Future work employing conditional transfer entropy frameworks that explicitly control for such common factors would provide a more stringent and rigorous test of the causal interpretation advanced here.

## Figures and Tables

**Table 1:** Entropy weights of indicators in the Composite Influence Index.

Indicator	Information Entropy	Redundancy	Weight	Weight (%)
Betweenness Centrality ($w_{1}$)	0.7610	0.2390	0.4028	40.28%
Reverse PageRank ($w_{2}$)	0.7894	0.2106	0.3549	35.49%
Out-Degree Centrality ($w_{3}$)	0.8562	0.1438	0.2423	24.23%
**Total**			1.0000	100%

**Table 2:** Missing value statistics for key variables.

Variable	Total Obs.	Missing Obs.	Missing Rate (%)
House price	7104	329	4.63
Regional GDP	7104	262	3.69
Registered population	7104	823	11.59

**Table 3:** Descriptive statistics.

Variables	Obs	Mean	Std. Dev.	Min	Max	Frequency
Regional GDP	7104	18,978,246.53	33,693,636.64	179,307	472,190,000	Year
Registered population	7104	425.75	312.50	15.96	3416	Year
House price	7104	5052.77	5000.60	439	60,347	Year
Mean of regional GDP	296	18,978,246.53	26,056,313.86	1,457,804.17	216,390,833.3	Year
Mean of registered population	296	425.75	310.78	19.40	3293.99	Year
Mean of house price	296	5052.77	3443.37	1390.37	31,044.15	Year

**Table 4:** National Housing Price Spatial Spillover Network Topology Index.

Rank	City	OutDegree Centrality	Rank	City	Standardized Betweenness Centrality	Rank	City	Reverse PageRank Index	Rank	City	Composite Influence Index
1	Beijing	16	1	Shanghai	0.0116	1	Shanghai	0.0911	1	Shanghai	89.69
2	Chengdu	14	2	Fuzhou	0.0089	2	Fuzhou	0.0805	2	Fuzhou	69.15
3	Chongqing	10	3	Beijing	0.0063	3	Quanzhou	0.0691	3	Beijing	62.53
4	Shanghai	9	4	Quanzhou	0.0059	4	Shenzhen	0.0676	4	Quanzhou	50.61
5	Shenzhen	9	5	Chengdu	0.0036	5	Xiamen	0.0625	5	Chengdu	45.16
6	Guangzhou	8	6	Wuhan	0.0031	6	Beijing	0.0438	6	Shenzhen	43.35
7	Nanjing	8	7	Xiamen	0.0030	7	Chengdu	0.0303	7	Xiamen	41.67
8	Shenyang	7	8	Huanggang	0.0029	8	Chongqing	0.0165	8	Chongqing	28.70
9	Zhengzhou	7	9	Ezhou	0.0027	9	Changchun	0.0150	9	Changchun	21.71
10	Changsha	7	10	Heze	0.0024	10	Wuhan	0.0130	10	Nanjing	21.64

**Figure A1:**
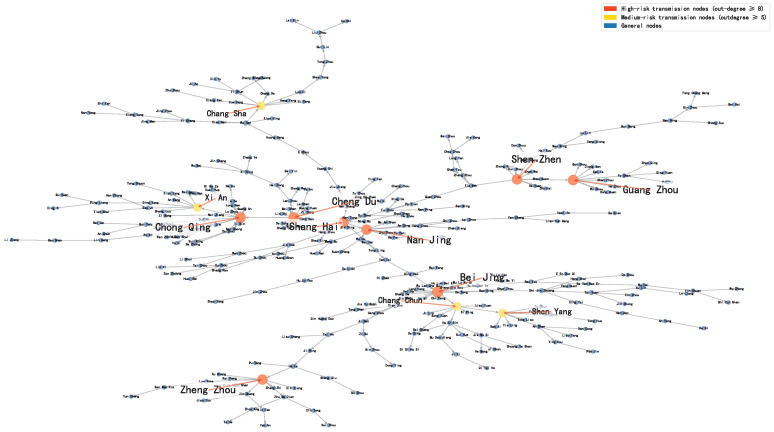
National housing price spatial spillover network under mean imputation. Red nodes identify cities whose direct transmission capacity is greater than or equal to 8, while yellow nodes identify cities whose direct transmission capacity falls within the range of 5 to 7.

**Figure A2:**
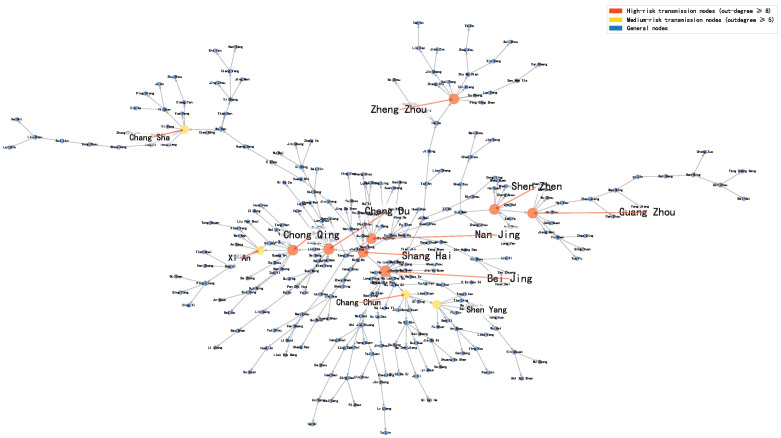
National housing price spatial spillover network under KNN imputation (k = 5). Red nodes identify cities whose direct transmission capacity is greater than or equal to 8, while yellow nodes identify cities whose direct transmission capacity falls within the range of 5 to 7.

**Figure A3:**
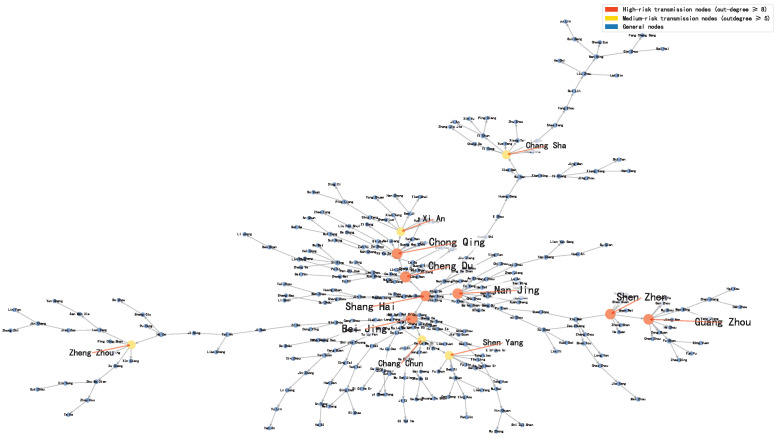
National housing price spatial spillover network under the alternative specification k=l=1, M=3 bins. Red nodes identify cities whose direct transmission capacity (out-degree) is greater than or equal to 8; yellow nodes identify cities whose out-degree falls within the range of 5 to 7; blue nodes represent general city nodes. Directed edges indicate the direction of housing price spillover transmission as extracted by the DMST algorithm.

**Figure A4:**
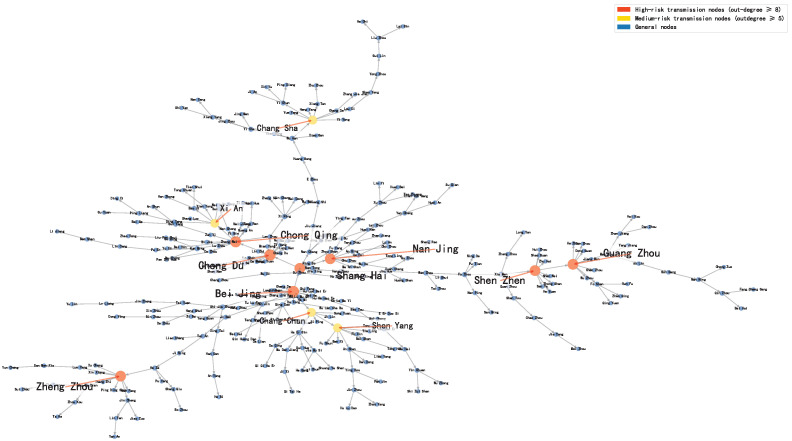
National housing price spatial spillover network under the alternative specification k=l=2, M=2 bins. Node classification and edge conventions follow Figure A3.

**Figure A5:**
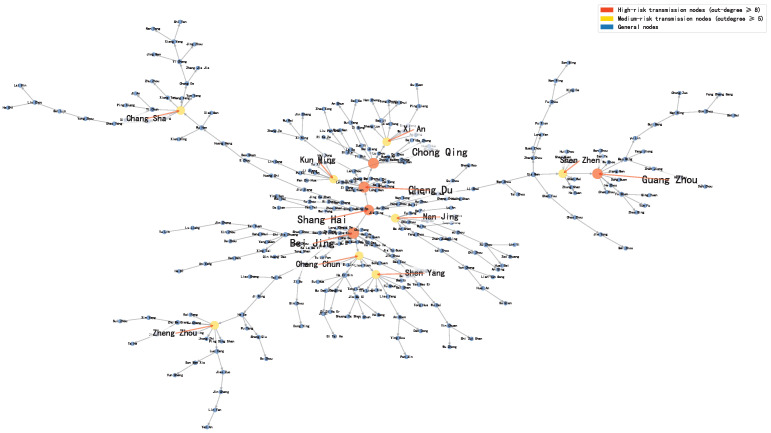
National housing price spatial spillover network under the alternative specification k=l=2, M=3 bins. Node classification and edge conventions follow Figure A3.

**Figure A6:**
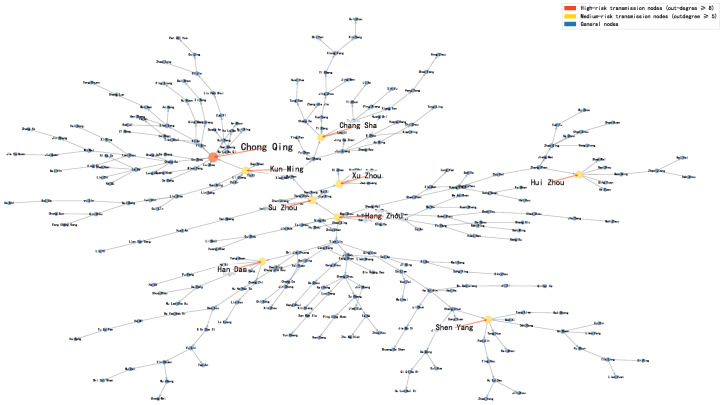
National housing price spatial spillover network under conventional TE-GM with k=l=1, M=2 bins. Red nodes identify cities whose direct transmission capacity (out-degree) is greater than or equal to 8; yellow nodes identify cities whose out-degree falls within the range of 5 to 7; blue nodes represent general city nodes. Directed edges indicate the direction of housing price spillover transmission as extracted by the DMST algorithm.

**Figure A7:**
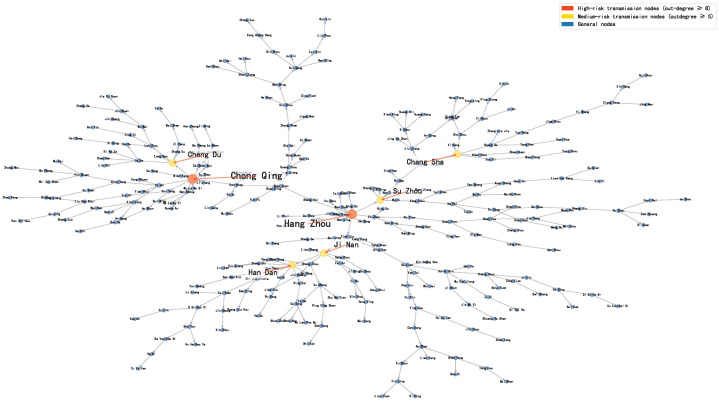
National housing price spatial spillover network under conventional TE-GM with k=l=1, M=3 bins. Node classification and edge conventions follow Figure A6.

**Figure A8:**
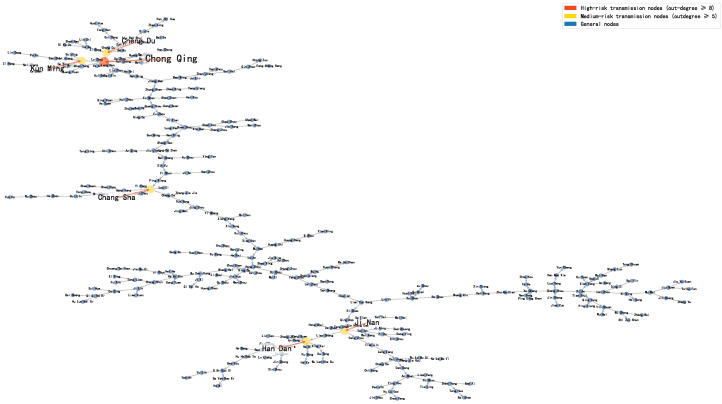
National housing price spatial spillover network under conventional TE-GM with k=l=2, M=2 bins. Node classification and edge conventions follow Figure A6.

**Figure A9:**
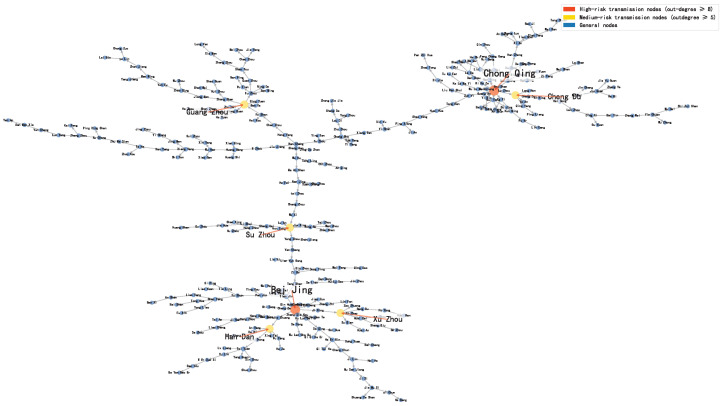
National housing price spatial spillover network under conventional TE-GM with k=l=2, M=3 bins. Node classification and edge conventions follow Figure A6.

## Data Availability

The data presented in this study are available on request from the corresponding author. Housing price data were retrieved from the Anjuke real estate platform (https://anjuke.com/ accessed on 1 September 2024). Regional GDP and registered population data were obtained from the China Statistical Yearbook (https://www.stats.gov.cn/sj/ndsj/ accessed on 1 September 2024).

## Abbreviations

The following abbreviations are used in this manuscript:

TE	Transfer Entropy
CBEDTE	Correlation-Dependent Balanced Estimation of Diffusion Transfer Entropy
CBEDE	Correlation-Dependent Balanced Estimation of Diffusion Entropy
BEDE	Balanced Estimation of Diffusion Entropy
GM	Gravity Model
DMST	Directed Minimum Spanning Tree
TSI	Total Spillover Index
CII	Composite Influence Index
VAR	Vector Autoregression
RMT	Random Matrix Theory
ECM	Error Correction Model
SADF	Supremum Augmented Dickey–Fuller
TVP-VAR	Time-Varying Parameter VAR
GDP	Gross Domestic Product

## Appendix A. Sensitivity Analysis of Missing Value Imputation Methods

To assess whether the main findings are sensitive to the choice of missing value imputation strategy, we replicate the national housing price spatial spillover network analysis using two alternative imputation methods: mean imputation (city-level temporal mean) and K-Nearest Neighbors (KNN) imputation with k=5, where neighbors are identified based on geographic province membership and GDP level similarity. Figure A1 and Figure A2 present the national spillover networks derived under mean imputation and KNN imputation respectively, which can be compared directly with Figure 5 in the main text.

The core structural features are highly consistent across all three imputation methods. In all cases, Beijing, Shanghai, Chengdu, and Chongqing retain their roles as dominant radiation hubs with elevated out-degree centrality, and the hierarchical ripple-effect architecture with eastern coastal cities at the apex is preserved throughout. These results confirm that the main findings reported in this study are robust to the treatment of missing observations.

## Appendix B. Robustness Checks: Alternative *CBEDTE* Parameter Specifications

To assess the sensitivity of the main empirical findings to the choice of hyperparameters in the CBEDTE estimator, we replicate the national housing price spatial spillover network analysis under three alternative parameter configurations: (i) k=l=1, M=3 bins; (ii) k=l=2, M=2 bins; and (iii) k=l=2, M=3 bins. These specifications differ from the baseline (k=l=1, M=2) in either the lag order governing the dependence structure of the source and target sequences, the coarseness of the value-range partitioning, or both. The resulting directed minimum spanning tree (DMST) networks are displayed in Figure A3, Figure A4 and Figure A5.

Across all three alternative specifications, the backbone topology of the spillover network remains qualitatively consistent with the baseline results reported in Figure 5 of the main text. In each case, the network exhibits the characteristic hierarchical ripple-effect architecture, with eastern coastal cities and Beijing sustaining their roles as dominant radiation hubs. The core-periphery unidirectional radiation structure is preserved without exception. Minor differences in the precise composition of secondary hub cities are observed across specifications, reflecting the sensitivity of peripheral connections to bin resolution and lag order, but these do not affect any substantive conclusion of the study. The robustness of the core findings across all four specifications (baseline and three alternatives) provides confidence that the results are not an artefact of a particular CBEDTE parameterization.

## Appendix C. Empirical Comparison Between *CBEDTE* and Conventional Transfer Entropy

To empirically validate the methodological advantage of the proposed CBEDTE under finite-length sequence conditions, we provide a direct comparison with conventional Transfer Entropy (TE). The comparison framework is kept perfectly consistent: CBEDTE and TE are respectively used as the causal measure, embedded into the gravity model (GM) to construct the inter-city housing price spillover intensity matrix, and the Directed Minimum Spanning Tree (DMST) algorithm is employed to extract the core spillover backbone. The only difference between the two methods lies in the choice of the information flow estimator.

Under identical parameter settings, the housing price spillover network constructed by TE-GM (Figure A6) identifies only Chong Qing as a high-risk spillover node, with secondary-risk spillover nodes including Han Dan, Chang Sha, Kun Ming, Xu Zhou, Su Zhou, Hang Zhou, Hui Zhou, and Shen Yang, collectively exhibiting a localized radiation pattern centered on Chong Qing as a single core. In contrast, the housing price spillover network constructed by CBEDTE-GM (Figure 5) reveals a markedly different structure: high-risk spillover nodes encompass seven cities, namely Bei Jing, Cheng Du, Chong Qing, Shang Hai, Nan Jing, Shen Zhen, and Guang Zhou, forming a multi-centric radiation system, while secondary-risk spillover nodes include Chang Sha, Xi An, Zheng Zhou, Shen Yang, and Chang Chun, which are widely distributed across central, western, and northeastern China. Regarding the ripple effect, the TE-GM network exhibits only limited diffusion from Chongqing to a subset of secondary cities; thus, the ripple effect remains indistinct. In contrast, the CBEDTE-GM network fully demonstrates the ripple effect of housing price spillovers. This comparison demonstrates that, under identical parameter settings, conventional TE causal identification suffers from estimation bias inherent to finite-length sequences, rendering it incapable of accurately extracting core spillover nodes and hierarchical transmission pathways. By mitigating the estimation bias of finite-length sequences, CBEDTE enables a more reliable identification of multi-centric radiation structures and complete ripple effects under the same parameter configuration, thereby providing a more robust foundation for network construction in housing price spatial spillover research.

To avoid the contingency of parameter selection on the comparison results, this paper examines four sets of parameter combinations: lag order k=l=1 with M=3 bins, k=l=2 with M=2 bins, and k=l=2 with M=3 bins. Figure A7, Figure A8 and Figure A9 present the national housing price spatial spillover networks constructed by the conventional TE-GM model under the above three parameter sets, respectively. Compared with the CBEDTE-GM network shown in Figure 5 of the main text, the TE-GM networks exhibit systematic deficiencies in the following two key dimensions.

First is the failure to effectively identify key city nodes. In the CBEDTE-GM network (Figure 5 of the main text), economically and geographically core cities such as Bei Jing, Cheng Du, Chong Qing, Shang Hai, Nan Jing, Shen Zhen, and Guang Zhou are clearly presented as high-radiation hub nodes. In contrast, among the four TE-GM networks, only Beijing and Chongqing emerge as significant radiation hubs among the above cities, while the radiation roles of Cheng Du, Shang Hai, Nan Jing, Shen Zhen, and Guang Zhou are obscured. Meanwhile, as shown in Figure A6, Figure A7, Figure A8 and Figure A9, the TE-GM networks identify some secondary hub cities that do not possess significant advantages in terms of population size, housing price level, or gross regional product, yet are misidentified as key nodes (e.g., “Hui Zhou”, “Han Dan”, “Kun Ming”). This indicates that conventional TE not only omits genuine core cities but also produces erroneous identification of key nodes under finite-sample conditions.

Second, the hierarchical spillover structure is obscured. The distinctive “ripple effect” hierarchical structure of China’s housing price spatial spillover is unrecognizable in all four TE-GM networks, which exhibit a non-hierarchical network topology. In contrast, the CBEDTE-GM network (Figure 5 of the main text) fully preserves this hierarchical structure, with clear core nodes and spillover paths that possess explicit geographic and economic rationality.

The above comparative results demonstrate that, under the finite-sample condition of 24 annual observations, the conventional TE estimator suffers from core node identification bias and obscuration of the hierarchical structure due to finite-sample bias, regardless of the parameter combination employed. In contrast, CBEDTE corrects for systematic bias through correlation-dependent balanced entropy estimation, significantly improving the clarity, economic interpretability, and hierarchical integrity of the network structure. This comparison validates the methodological necessity of adopting CBEDTE as the core causal identification tool in this study.
